# New Insights into 4-Anilinoquinazolines as Inhibitors of Cardiac Troponin I–Interacting Kinase (TNNi3K)

**DOI:** 10.3390/molecules25071697

**Published:** 2020-04-07

**Authors:** Christopher R. M. Asquith, Tuomo Laitinen, Carrow I. Wells, Graham J. Tizzard, William J. Zuercher

**Affiliations:** 1 Department of Pharmacology, School of Medicine, University of North Carolina at Chapel Hill, Chapel Hill, NC 27599, USA; 2 Structural Genomics Consortium, UNC Eshelman School of Pharmacy, University of North Carolina at Chapel Hill, Chapel Hill, NC 27599, USA; carrow.wells@unc.edu (C.I.W.); william.zuercher@unc.edu (W.J.Z.); 3 School of Pharmacy, Faculty of Health Sciences, University of Eastern Finland, 70211 Kuopio, Finland; tuomo.laitinen@uef.fi; 4 UK National Crystallography Service, School of Chemistry, University of Southampton, Highfield Campus, Southampton SO17 1BJ, UK; graham.tizzard@soton.ac.uk; 5 Lineberger Comprehensive Cancer Center, University of North Carolina at Chapel Hill, Chapel Hill, NC 27599, USA

**Keywords:** cardiac troponin I–interacting kinase (TNNi3K), 4-anilino-quinazolines, conformational flexibility, hinge binder, kinase inhibitor design

## Abstract

We report the synthesis of several related 4-anilinoquinazolines as inhibitors of cardiac troponin I–interacting kinase (TNNi3K). These close structural analogs of 3-((6,7-dimethoxyquinazolin-4-yl)amino)-4-(dimethylamino)-*N*-methylbenzenesulfonamide (GSK114) provide new understanding of structure–activity relationships between the 4-anilinoquinazoline scaffold and TNNi3K inhibition. Through a small focused library of inhibitors, we observed that the *N*-methylbenzenesulfonamide was driving the potency in addition to the more traditional quinazoline hinge-binding motif. We also identified a compound devoid of TNNi3K kinase activity due to the addition of a methyl group in the hinge binding region. This compound could serve as a negative control in the study of TNNi3K biology. Small molecule crystal structures of several quinazolines have been solved, supporting observations made about overall conformation and TNNi3K inhibition.

## 1. Introduction

Kinases have been successfully targeted with 52 kinase inhibitors approved by the FDA to date. These kinase inhibitor drugs tend to be predominantly multi-targeted tyrosine kinase inhibitors for the treatment of cancer [1,2]. However, with over 500 kinases in the human genome, kinase inhibitors have therapeutic potential that extends beyond cancer [3], and there remains vast untapped potential within the kinome for treating a wide range of human diseases [4].To identify which kinases play key roles beyond oncology, highly potent and selective inhibitors are required [2]. 

One example of less-studied kinase is cardiac troponin I-interacting kinase (TNNi3K or CARK), a member of the wider tyrosine-like kinase (TLK) family. TNNi3K is selectively expressed in heart tissues and has been linked to cardiac hypertrophy dilated cardiomyopathy, pressure overload-induced heart failure, and ischemia/reperfusion injury in an in-vivo neonatal rat model [5,6,7]. A TNNi3K knockout mouse exhibited reduced ischemic injury suggesting that deletion of TNNi3K is cardioprotective [5,6,7]. These studies suggest that TNNi3K inhibition could serve as a unique strategy for addressing acute ischemic injury and heart failure [5,6,7].

**Figure 1:**
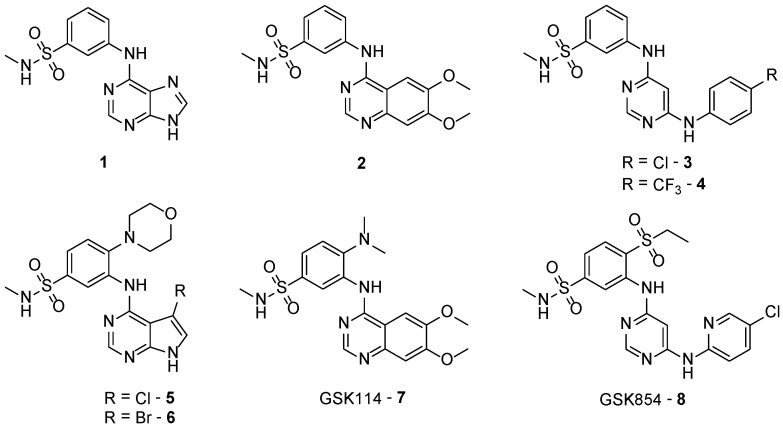
Previously reported TNNi3K inhibitors (**1**–**8**).

TNNi3K inhibitor development could serve the purpose of developing novel heart failure therapeutics and probing biological function of TNNi3K. Several TNNi3K inhibitor structures have been previously disclosed (**1**–**8**), each with some common structural themes (Figure 1) [8,9,10,11]. These include the structural element of a *N*-methylbenzenesulfonamide and a preference for a flat molecular conformation around the pyrimidine/quinazoline hinge binding moeity [8,9,10,11]. We now describe a small array of new inhibitors of TNNi3K based on GSK114 (**7**) to gain additional insights into the structural requirements for TNNi3K inhibition.

## 2. Results and Discussion 

### 2.1. Synthesis of Aniline **13**

**Scheme 1:**
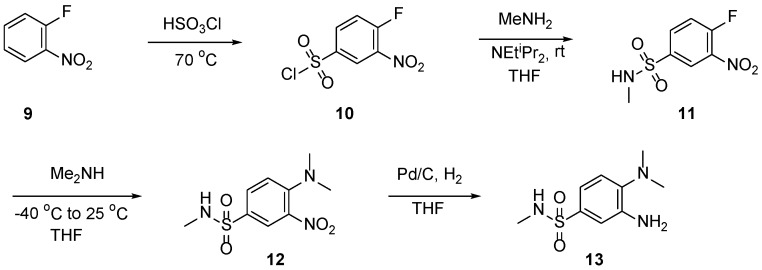
Synthetic route to access non-commercial aniline **13**.

To synthesize analogs of GSK114 (**7**) we first needed to access the previously reported aniline building block (Scheme 1) [8,9,10]. First 1-fluoro-2-nitrobenzene (**9**) was treated with neat chlorosulfonic acid at 0 °C and then refluxed for 2 h to afford the sulfonyl chloride derivative (**10**) in 69% yield (5 g scale). Next, **10** was then treated with methyl amine to afford the *N*-methylbenzenesulfonamide product (**11**) in 81% yield (5 g scale) followed by a fluoride displacement using dimethylamine affording the nitro compound (**12**) in good yield (84%). The nitro group was reduced with hydrogen and Pd/C to furnish the aniline (**13**) in quantitative yield.

### 2.2. Synthesis of 4-Anilinoquinazolines ***2**, **7**, **15**–**17** & **20***

**Scheme 2:**
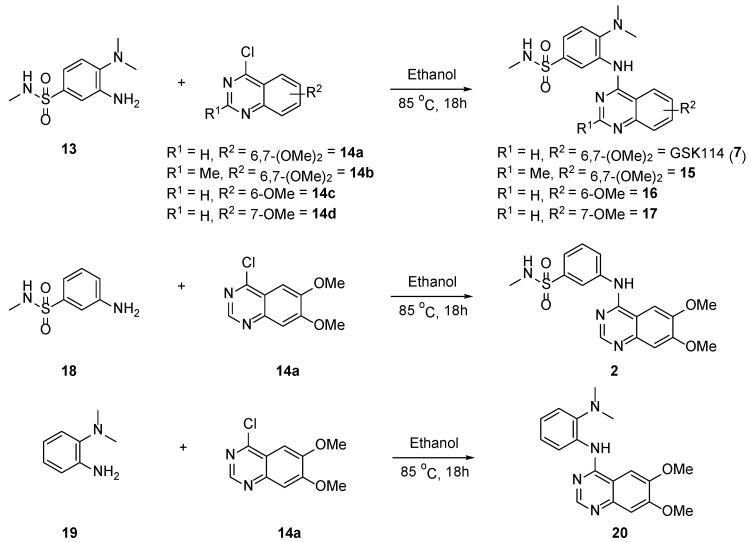
Synthetic route to access 4-anilinoquinazolines (**2**, **7**, **15**–**17** & **20**).

Aniline **13** was then reacted with the corresponding 6-bromo-4-chloroquinoline derivatives (**14a**–**d**) to afford 4-anilinoquinazolines (**7** and **15**–**17**) via a nucleophilic aromatic substitution reaction in good overall yields (53–67%) (Scheme 2) [12,13,14,15,16,17,18,19]. The two other derivatives (**2** and **20**) were afforded by reaction of commercially available anilines **18** and **19**, respectively, with 4-chloro-6,7-dimethoxyquinazoline (**14a**) via the same route as **7** and **15**–**17** to afford **2** in excellent yield (78%) and **20** in good yield (57%). 

### 2.3. Results of 4-Anilinoquinazolines ***2**, **7**, **15**–**17** & **20*** in TNNi3K Binding Assay

GSK114 (**7**) provided good baseline activity with an IC_50_ = 25 nM (Table 1) in an enzyme assay [10]. The incorporation of a methyl group in the hinge-binding region (**15**) led to complete loss of TNNi3K activity. The removal of the methoxy group in the 7-position (**16**) led to a decrease in potency by roughly 4-fold. Interestingly, removal of the other methoxy group at the 6-position (**17**) resulted in a 2-fold increase in potency. The removal of the dimethylamine substitution (**2**) on the aniline led to a nearly 5-fold loss of activity. However, removal of the *N*-methylbenzenesulfonamide (**20**) was more significant with an almost 250-fold drop in potency compared to GSK114 (**7**). 

### 2.4. KinomeScan^®^ of GSK114 (***7***) and ***15***

**Figure 2:**
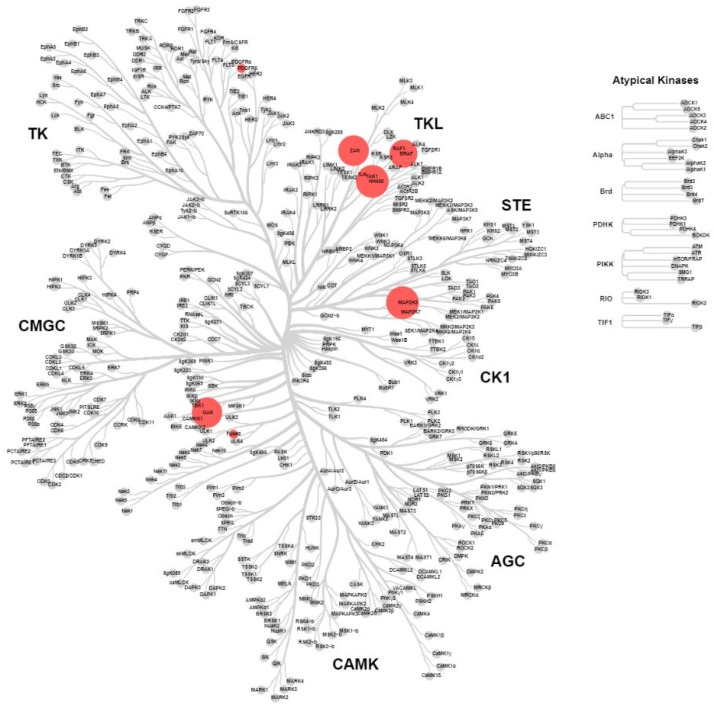
Representation of the human kinome based on sequence identity displaying KINOMEscan^®^ results for compound **7** screened at 1 μM. Red dots represent kinases inhibited by >80%, differentiated by size; wild-type human kinases inhibited >80% at 1 μM MEK5, 100%; STK36, 94%; ZAK, 93%; GAK, 92%; PDFRB, 91%; BRAF 84% and TNNi3K, IC_50_ = 25 nM [10] (see Supplementary Materials).

**Figure 3:**
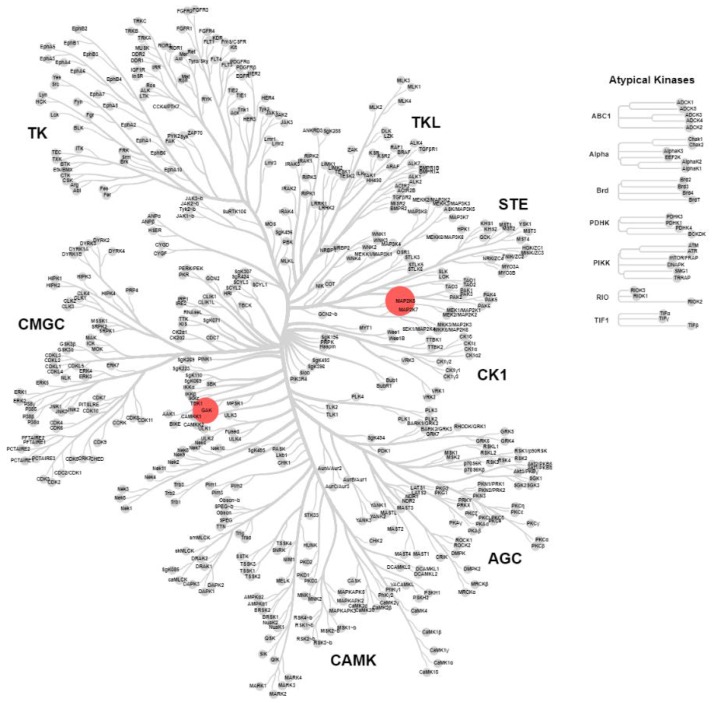
Representation of the human kinome based on sequence identity displaying KINOMEscan^®^ results for compound **15** screened at 1 μM. Red dots represent kinases inhibited by >75%, differentiated by size; kinases inhibited at 1 μM MEK5, 92% and GAK, 78% (see Supplementary Materials).

GSK114 (**7**) and **15** were submitted for a KINOMEscan^®^ assay to explore the selectivity profile across >400 human kinases at a compound concentration of 1 µM. The screen (Figure 2 and Figure 3) showed activity on only six kinases for compound **7** (with TNNi3K added from Lawhorn BG et al. [10]: MEK5, STK36, ZAK, GAK, PDFRB, BRAF and TNNi3K [10]) and two kinases (MEK5 and GAK) for **15**. GSK114 (**7**) is a narrow-spectrum TNNi3K inhibitor but has some potent off-targets including GAK which has been previously reported for this chemotype [12,13,14,15]. GAK and MEK5 were also identified in the screening of the hinge-blocked analog (**15**), albeit significantly weaker activity than in the case of **7**. If utilized together this set of compounds could be useful to investigate TNNi3K biology.

### 2.5. Modelling of Inhibitors in TNNi3K

**Figure 4:**
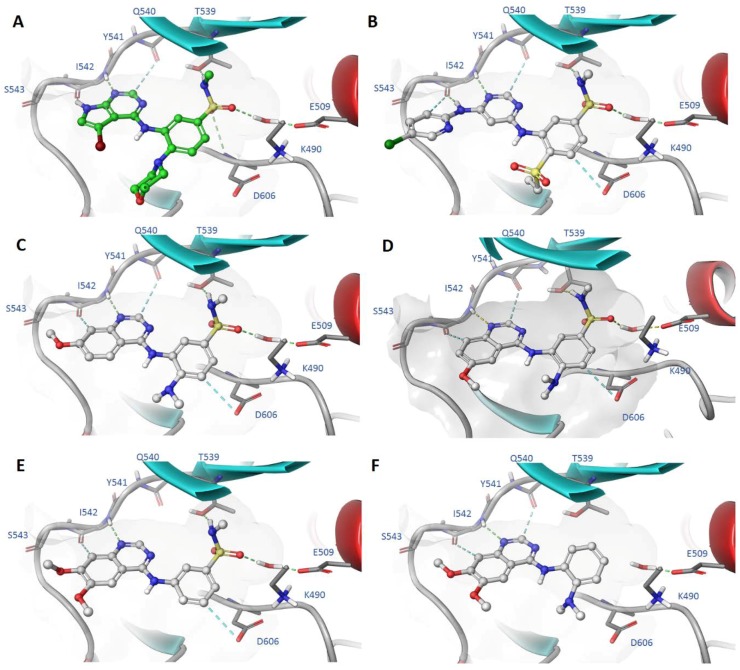
Modelling of compounds **6** (**A**), **8** (**B**), **17** (**C**), **16** (**D**), 2 (**E**) and **20** (**F**) docked in the TNNi3K ATP binding site (PDB: 4YFF).

The molecular modelling demonstrates that the *N*-methylbenzenesulfonamide interaction formed through a water-mediated bridge with the carboxylic acid of E509 and with the alcohol of T539 provides significant binding affinity in addition to the hinge contact (Figure 4). Literature compounds **6** and **8** (Figure 4A,B) show a binding pose mediated by the hinge-binding motif, but also by the *N*-methyl-benzenesulfonamide as a main interaction. The same binding interactions are also observed in compounds **17**, **16**, **2** in Figure 4C–E respectively, with the dimethylamine assisting in the conformational preference of the optimal inhibitor. Interestingly, despite the hinge binder being present in compound **20**, the potency was significantly weaker, and this was supported by the modelling of **20** showing a loose, non-coordinated interaction of the aniline portion of the molecule in Figure 4F. 

**Figure 5:**
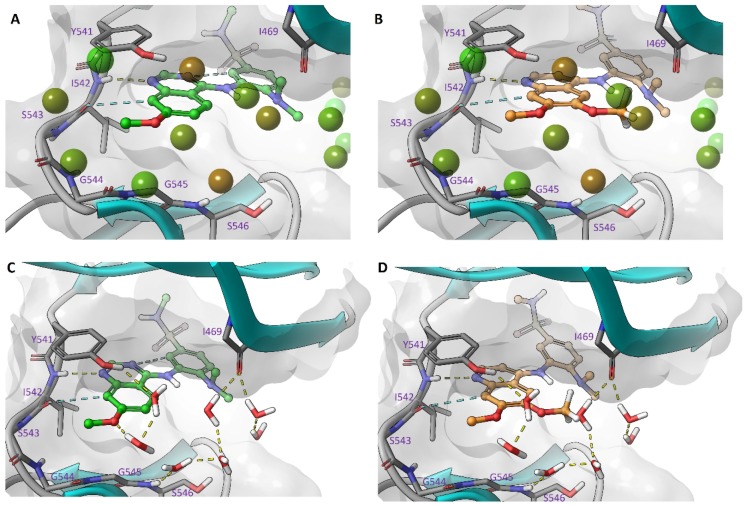
Docked **17** and **7** in the TNNi3K ATP binding site (PDB: 4YFF). WaterMap of **17** (**A**) and **7** (**B**) showing water molecules as spheres and WaterMap of **17** (**C**) and **7** (**D**) showing water molecules with hydrogen bonding networks.

One interesting observation of this focused library was the slight improvement in potency of **17** over **7** despite the very small difference with removal of just one methoxy group. In order to investigate this effect and rationalize the improved potency we simulated the bound water networks of **17** and **7** (Figure 5) and found less disruption to the solvent network of **17** compared to **7**. In the absence of direct interactions outside of simple Van der Waals contacts this provides a logical rationale for the increased potency observed with **17** [20].

### 2.6. Small Molecule Crystal Structures of **16**, **17** and **20**

**Figure 6:**
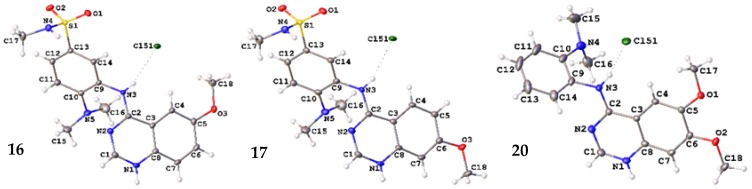
Molecular structure of **16**H^+^Cl^−^, **17**H^+^Cl^−^ and **20**H^+^Cl^−^ showing atomic labeling and displacement ellipsoids at a 50% probability level.

A crystallographic analysis revealed **16**, **17** and **20** crystallized as chloride salts (Figure 6) with **16** and **17** crystallizing in the triclinic *P-1* space group and **20** crystallizing in the monoclinic *I2/a* space group. Each of the molecules comprise a planar quinazoline moiety and substituted aniline moiety. The quinazoline moiety, N1-N2 and C1-C8, exhibit root mean square (r.m.s) deviations between 0.011 Å (**20**) and 0.034 Å (**16**) with the maximum deviation from the plane being between 0.019 Å (**20**) and 0.0057 Å (**16**) for the aniline N-bonded C2 atom in all molecules. The r.m.s. deviation of the aniline moieties (excluding the dimethylamine and *N*-methylbenzenesulfonamide substituents) is between 0.021 Å (**16**) and 0.059 Å (**20**) with the aniline N-atom bonded C9 displaying the maximum deviation of 0.029 Å (**16**) or 0.031 Å (**17**) or for **20**, the aniline N-atom N3 (0.093 Å). The dihedral angles between the two aforementioned planes are 55.75(3)° (16), 56.42(5)° (17) and 64.57(3)° (20). The C2-N3 bond distances of 1.3423(13) Å (**16**), 1.344(2) Å (**17**) and 1.3309(12) Å (**20**) are indicative of a double bond character in this bond consistent with conjugation in the quinazoline moieties. All other bond distances and angles are within expected limits.

**Figure 7:**
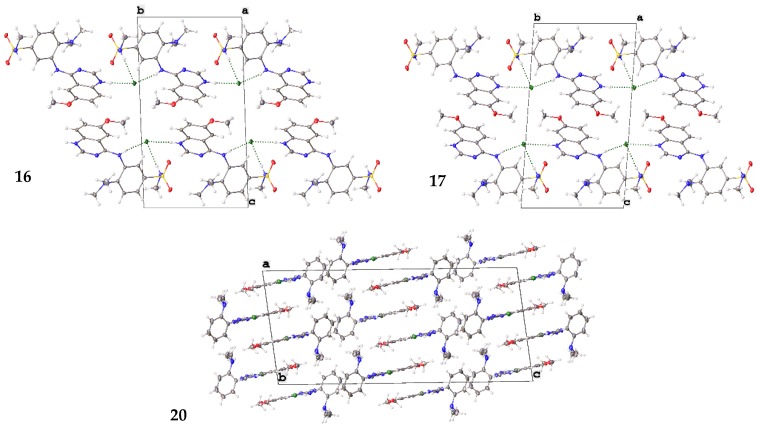
Unit cell contents of **16**H^+^Cl^−^, **17**H^+^Cl^−^ and **20**H^+^Cl^−^ shown in projection down the *a*-axis (**16**, **17**) or *b*-axis (**20**). Hydrogen bonds are shown as dashed green lines.

The chloride anion is integral to the solid-state structures of each of the compounds (Figure 7). The structures of **16** and **17** are isostructural to a reasonable approximation and in these structures stacks, parallel to the a-axis, of protonated cationic molecules are hydrogen bonded to Cl^−^ via the quinazoline N-H^+^ (N-H^+…^Cl^−^ (3.0741(10) Å (**16**), 3.1185(17) Å (**17**)), aniline N-H (N-H^…^Cl^−^ (3.128(1) Å (**16**), 3.1896(16) Å (**17**)) and the *N*-methylbenzenesulfonamide N-H (N-H^…^Cl^−^ (3.2989(11) Å (**16**), 3.2747(18) Å (**17**)). These hydrogen bonds link neighboring molecules in each stack with molecules in adjacent stacks via each chloride anion to form two-dimensional layers which stack via π-π interactions to form the structures. The structure of **20** comprises one-dimensional ‘tapes’ of **20**H^+^ hydrogen bonded to Cl^−^ via the quinazoline N-H^+^ and aniline N-H respectively (N-H^+…^Cl^−…^H-N (2.9732(9) Å, 3.1444(9) Å)) parallel to the *b*-axis. These tapes form two-dimensional, interleaved layers via π-π interactions parallel to the a-axis which close-pack to form the structure.

## 3. Experimental Section 

### 3.1. Chemistry

All reactions were performed using flame-dried round-bottomed flasks or reaction vessels. Where appropriate, reactions were carried out under an inert atmosphere of nitrogen with dry solvents, unless otherwise stated. Yields refer to chromatographically and spectroscopically pure isolated yields. Reagents were purchased at the highest commercial quality and used without further purification. Reactions were monitored by thin-layer chromatography carried out on 0.25 mm E. Merck silica gel plates (60_F-254_) using ultraviolet light as the visualizing agent. NMR spectra were recorded on a Varian Inova 400 spectrometer (Varian, Palo Alto, CA, USA) and were calibrated using residual undeuterated solvent as an internal reference (CDCl_3_: ^1^H NMR = 7.26, ^13^C NMR = 77.16). The following abbreviations or combinations thereof have been used to explain the multiplicities observed: s = singlet, d = doublet, t = triplet, q = quartet, m = multiplet, br = broad. Liquid chromatography (LC) and high-resolution mass spectra (HRMS) were recorded on a ThermoFisher hybrid LTQ FT (ICR 7T) (ThermoFisher, Waltham, MA, USA). The University of Southampton (Southampton, UK) small molecule X-ray facility collected and analyzed all X-ray diffraction data. Compounds **10** and **11** were prepared as previously described [8].

*4-(dimethylamino)-N-methyl-3-nitrobenzene-1-sulfonamide* (**12**) was obtained as a yellow solid (465 mg, 1.793 mmol, 84%). MP 132–134 °C; ^1^H NMR (400 MHz, DMSO-*d*_6_) δ 8.09 (d, *J* = 2.3 Hz, 1H), 7.75 (dd, *J* = 9.1, 2.3 Hz, 1H), 7.32 (d, *J* = 9.1 Hz, 1H), 2.93 (s, 6H), 2.40 (s, 3H). HRMS *m*/*z* [M + H]^+^ calculated for C_9_H_14_N_3_O_4_S: 260.0705, found 260.0696, LC t_R_ = 4.00 min, >98% purity, consistent with previously reported results [8].

*3-amino-4-(dimethylamino)-N-methylbenzene-1-sulfonamide* (**13**) was obtained as a purple solid (442 mg, 1.928 mmol, 100%). MP 66–68 °C; ^1^H NMR (400 MHz, DMSO-*d*_6_) δ 7.15–7.04 (m, 2H), 7.00 (d, *J* = 8.2 Hz, 1H), 6.93 (dd, *J* = 8.2, 2.2 Hz, 1H), 5.14 (s, 2H), 2.61 (s, 6H), 2.38 (d, *J* = 5.0 Hz, 3H). HRMS *m*/*z* [M + H]^+^ calculated for C_9_H_16_N_3_O_2_S: 230.0963, found 230.0956, LC t_R_ = 2.77 min, >98% purity consistent with previously reported results [8].

*3-((6,7-dimethoxyquinazolin-4-yl)amino)-4-(dimethylamino)-N-methylbenzenesulfonamide* (**7**) was obtained as a yellow solid (98.5 mg, 0.236 mmol, 53%). MP 244–246 °C; ^1^H NMR (400 MHz, DMSO-*d*_6_) δ 11.46 (s, 1H), 8.75 (s, 1H), 8.34 (s, 1H), 7.70–7.55 (m, 2H), 7.40 (s, 1H), 7.32 (q, *J* = 5.0 Hz, 1H), 7.22–7.15 (m, 1H), 3.98 (s, 6H), 2.79 (s, 6H), 2.40 (d, *J* = 5.0 Hz, 3H). ^13^C NMR (100 MHz, DMSO-*d*_6_) δ 159.0, 156.4, 151.9, 150.3, 148.8, 135.4, 129.2, 128.6, 126.8, 125.9, 117.7, 107.2, 103.9, 99.7, 56.9, 56.5, 42.2 (2C, s), 28.7. HRMS *m*/*z* [M + H]^+^ calculated for C_19_H_24_N_5_O_4_S: 418.1549, found 418.1540, LC t_R_ = 3.04 min, >98% purity consistent with previously reported results [10].

*3-((6,7-dimethoxy-2-methylquinazolin-4-yl)amino)-4-(dimethylamino)-N-methylbenzenesulfonamide* (**15**) was obtained as a yellow solid (101 mg, 0.235 mmol, 56%). MP 252-254 °C; ^1^H NMR (400 MHz, DMSO-*d*_6_) δ 11.23 (s, 1H), 8.28 (s, 1H), 7.67 (d, *J* = 2.3 Hz, 1H), 7.63 (dd, *J* = 8.7, 2.3 Hz, 1H), 7.39 (s, 1H), 7.32 (q, *J* = 5.0 Hz, 1H), 7.21 (d, *J* = 8.8 Hz, 1H), 3.98 (s, 6H), 2.80 (s, 6H), 2.52 (s, 3H), 2.44 (d, *J* = 5.0 Hz, 3H). ^13^C NMR (100 MHz, DMSO-*d*_6_) δ 159.0, 158.4, 156.2, 151.6, 149.7, 136.0, 129.0, 128.5, 126.5, 125.9, 117.8, 105.7, 103.8, 99.1, 56.8, 56.4, 42.0 (2C, s), 28.7, 21.9. HRMS *m*/*z* [M + H]^+^ calculated for C_20_H_26_N_5_O_4_S: 432.1706, found 432.1700, LC t_R_ = 3.25 min, >98% purity. 

*4-(dimethylamino)-3-((6-methoxyquinazolin-4-yl)amino)-N-methylbenzenesulfonamide* (**16**) was obtained as a yellow solid (127 mg, 0.387 mmol, 64%). MP 224–226 °C; ^1^H NMR (400 MHz, DMSO-*d*_6_) δ 11.60 (s, 1H), 8.81 (s, 1H), 8.35 (d, *J* = 2.6 Hz, 1H), 7.94 (d, *J* = 9.2 Hz, 1H), 7.76 (dd, *J* = 9.2, 2.6 Hz, 1H), 7.68 (d, *J* = 2.2 Hz, 1H), 7.65 (dd, *J* = 8.6, 2.3 Hz, 1H), 7.33 (q, *J* = 5.0 Hz, 1H), 7.22 (d, *J* = 8.7 Hz, 1H), 3.98 (s, 3H), 2.82 (s, 6H), 2.43 (d, *J* = 4.9 Hz, 3H). ^13^C NMR (100 MHz, DMSO-*d*_6_) δ 159.9, 159.1, 151.8, 149.1, 133.6, 129.3, 128.5, 127.0, 126.9, 125.7, 121.7, 117.8, 114.6, 104.3, 56.6, 42.2 (2C, s), 28.7. HRMS *m*/*z* [M + H]^+^ calculated for C_18_H_22_N_5_O_3_S: 388.1443, found 388.1434, LC t_R_ = 3.08 min, >98% purity consistent with previously reported results [10].

*4-(dimethylamino)-3-((7-methoxyquinazolin-4-yl)amino)-N-methylbenzenesulfonamide* (**17**) was obtained as a mustard solid (133 mg, 0.344 mmol, 67%). MP 216–219 °C; ^1^H NMR (400 MHz, DMSO-*d*_6_) δ 11.69 (s, 1H), 8.86 (d, *J* = 9.2 Hz, 1H), 8.82 (s, 1H), 7.73–7.57 (m, 2H), 7.49 (dd, *J* = 9.3, 2.5 Hz, 1H), 7.42 (d, *J* = 2.5 Hz, 1H), 7.36 (q, *J* = 5.1 Hz, 1H), 7.23–7.18 (m, 1H), 3.98 (s, 3H), 2.80 (s, 6H), 2.42 (d, *J* = 4.5 Hz, 3H).^13^C NMR (100 MHz, DMSO-*d*_6_) δ 164.9, 159.9, 151.8, 150.9, 140.8, 129.3, 128.5, 127.0, 126.9, 125.8, 119.2, 117.9, 107.2, 100.2, 56.4, 42.2 (2C, s), 28.7. HRMS *m*/*z* [M + H]^+^ calculated for C_18_H_22_N_5_O_3_S: 388.1443, found 388.1436, LC t_R_ = 3.07 min, >98% purity. 

*3-((6,7-dimethoxyquinazolin-4-yl)amino)-N-methylbenzenesulfonamide* (**2**) was obtained as a beige solid (195 mg, 0.521 mmol, 78%). MP 246–248 °C; ^1^H NMR (400 MHz, DMSO-*d*_6_) δ 11.77 (s, 1H), 8.85 (s, 1H), 8.44 (s, 1H), 8.16 (q, *J* = 1.4 Hz, 1H), 8.09–8.02 (m, 1H), 7.78–7.63 (m, 2H), 7.60 (q, *J* = 5.0 Hz, 1H), 7.40 (s, 1H), 4.01 (s, 3H), 3.96 (s, 3H), 2.47 (d, *J* = 4.9 Hz, 3H). ^13^C NMR (100 MHz, DMSO-*d*_6_) δ 158.2, 156.4, 150.3, 148.5, 139.8, 137.7, 135.7, 129.6, 128.4, 123.9, 122.7, 107.4, 104.2, 99.6, 57.1, 56.5, 28.8. HRMS *m*/*z* [M + H]^+^ calculated for C_17_H_19_N_4_O_4_S: 375.1127, found 375.1112, LC t_R_ = 2.91 min, >98% purity consistent with previously reported results [9].

*N^1^-(6,7-dimethoxyquinazolin-4-yl)-N^2^,N^2^-dimethylbenzene-1,2-diamine* (**20**) was obtained as a beige solid (124 mg, 0.381 mmol, 57%). MP 224–226 °C; ^1^H NMR (400 MHz, DMSO-*d*_6_) δ 11.22 (s, 1H), 8.69 (s, 1H), 8.33 (s, 1H), 7.44 (s, 1H), 7.34 (ddd, *J* = 15.6, 7.7, 1.6 Hz, 2H), 7.22 (d, *J* = 8.1 Hz, 1H), 7.08 (td, *J* = 7.5, 1.4 Hz, 1H), 3.99 (d, *J* = 2.6 Hz, 6H), 2.68 (s, 6H). ^13^C NMR (100 MHz, DMSO-*d*_6_) δ 159.0, 156.1, 150.1, 148.7, 135.9, 129.1 (2C, s), 129.0 (2C, s), 128.2, 122.0, 118.9, 107.3, 103.9, 100.2, 56.8, 56.4, 43.3 (2C, s). HRMS *m*/*z* [M + H]^+^ calculated for C_18_H_21_N_4_O_2_: 325.1665, found 325.1657, LC t_R_ = 2.60 min, >98% purity. 

### 3.2. Mass Spectrometry

Samples were prepared as previously described (see Supplementary Materials) [19].

### 3.3. Molecular Modelling

Prior to modelling studies, the structure of the TNNi3K X-ray structure (PDB: 4YFF) [8] was pre-processed by stepwise manner using the protein preparation wizard tool of Schrödinger Suite 2019-3 (Protein Preparation Wizard uses modules: Epik; Impact and Prime, Schrödinger, LLC, New York, NY, 2019). Preliminary structural inspections of the protein structures, including visualization of electronic density maps of the protein ligand complexes, were done online at the PDB site (https://www.ebi.ac.uk/pdbe/entry/pdb/4yfi). Consequently, the conserved hinge-binding bridged water at the site of back pocket was left in a place, as suggested by original publication [8]. Structures of small molecule ligands were parametrized and minimized using LigPrep module (LigPrep, Schrödinger, LLC, New York, NY, 2019). Molecular docking studies were computed using Glide module of Schrödinger employing XP-setting with softened Van der Waals potential (0.5 in place of default 0.8). A hydrogen bond constraint was added to hinge residue ILE542 to improve convergence of docking poses.

A hydration site analysis was calculated using WaterMap functionality of Schrödinger suite 2019-4. Analysis was computed for empty protein (PDB: 4YFF) and for IFD docking pose of compound **7** and **17** retained in ATP-binding site. 

### 3.4. Crystallography

For each compound a suitable crystal was selected and mounted on a MITIGEN holder (MiTeGen, Ithaca, NY, USA) in perfluoroether oil on a Rigaku FRE+ diffractometer equipped with VHF Varimax confocal mirrors and an AFC12 goniometer and HyPix 6000 detector. The crystal was kept at a steady *T* = 100(2) K during data collection. The structure was resolved by the ShelXT [21] structure solution program using the using dual methods solution method and using Olex2 [22] as the graphical interface. The model was refined with version 2018/3 of ShelXL [23], using full matrix least squares minimization on *F*^2^ minimization. All non-hydrogen atoms were refined anisotropically. Hydrogen atom positions were calculated geometrically and refined using the riding model, except for those bonded to N-atoms which were located in the difference map and refined with a riding model. The data for **17**H^+^Cl^−^ were processed as a 2-component non-merohedral twin (rotation (UB1, UB2) = 179.7789° around 0.66 0.74 0.13 (rec.) 0.93 0.37 −0.00 (dir) lattice vectors).

Crystal data for **16**H^+^Cl^−^, *Mr* = 423.91; triclinic; *P-1* (No. 2); a = 6.23720(10)Å; b = 9.54880(10)Å; c = 17.2769(3)Å; *α* = 91.5310(10)°, *β* = 92.682(2)°, *γ* = 99.235(2)°, *V* = 1013.91(3) Å^3^, *T* = 100(2) K, *Z* = 2, *Z’* = 1, μ(Mo K_α_) = 0.321 mm^−1^, 27,000 reflections measured, 5645 unique (*R_int_* = 0.0286) which were used in all calculations. The final *wR2* was 0.0860 (all data) and *R1* was 0.0325 (I > 2(I)).

Crystal data for **17**H^+^Cl^−^, *Mr* = 423.91, triclinic, *P-1* (No. 2), a = 6.1936(2)Å, b = 9.6619(3)Å, c = 17.1723(5)Å, *α* = 85.698(2)°, *β* = 87.900(2)°, *γ* = 79.989(2)°, *V* = 1008.84(5)Å^3^, *T* = 100(2) K, *Z* = 2, *Z’* = 1, *μ*(Mo K_α_) = 0.322 mm^−1^, 9229 reflections measured, 9229 unique, which were used in all calculations. The final *wR2* was 0.1356 (all data) and *R1* was 0.0466 (I > 2(I)).

Crystal data for **20**H^+^Cl^−^, *Mr* = 360.84, monoclinic, *I2/a* (No. 15), a = 13.3145(2)Å, b = 9.38030(10)Å, c = 29.4816(5)Å, *α* = 97.253(2)°, *β* = *γ* = 90°, *V* = 3652.61(9)Å^3^, *T* = 100(2) K, *Z* = 8, *Z’* = 1, *μ*(Mo K_α_) = 0.228 mm^−1^, 47,137 reflections measured, 5099 unique (*R_int_* = 0.0264) which were used in all calculations. The final *wR2* was 0.0915 (all data) and *R1* was 0.0330 (I > 2(I)).

### 3.5. KinomeScan^®^ Assay

KinomeScan^®^ Assay on **7** and **15** was performed as previously described by EuroFins DiscoverX [24].

## 4. Conclusions 

We have demonstrated a robust way to access the 4-anilinoquinazoline scaffold in good yield supported by previous literature [12,13,14,15,16,17,18,19]. We report the synthesis of several related 4-anilinoquinazolines as inhibitors of cardiac troponin I–interacting kinase (TNNi3K). These close structural analogs of GSK114 (**7**) provide new understanding of structure–activity relationships between the 4-anilinoquinazoline scaffold and TNNi3K inhibition. Further, an interesting lesson in kinase design was found: having the hinge binder present does not always guarantee a potent compound, as in the case of **2** vs **20**, where **20** without the *N*-methyl-benzenesulfonamide peripheral interaction is significantly weaker in TNNi3K inhibition. However, both interactions are required for a potent TNNi3K inhibitor. We also show that a simple hinge block with a methyl group (**15**) is enough to create a scaffold/negative control compound. We hope that the insights provide the medicinal chemist with an enhanced toolbox for kinase inhibitor design.

## Figures and Tables

**Table 1:** Results of 4-anilinoquinazolines **2**, **7**, **15–17** & **20** in the TNNi3K binding assay.

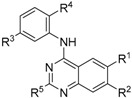						
Compound	R^1^	R^2^	R^3^	R^4^	R^5^	TNNi3K (K_d_) ^a^ (nM)
**7** (GSK114)	OMe	OMe	*N*-SO_2_NHMe	NMe_2_	H	25 ^b^
**15**	OMe	OMe	*N*-SO_2_NHMe	NMe_2_	Me	0% Inh. at 1 µM
**16**	OMe	H	*N*-SO_2_NHMe	NMe_2_	H	90
**17**	H	OMe	*N*-SO_2_NHMe	NMe_2_	H	11
**2**	OMe	OMe	*N*-SO_2_NHMe	H	H	120
**20**	OMe	OMe	H	NMe_2_	H	6200

^a^ binding assay (*n* = 1); ^b^ previously reported enzyme assay IC_50_ [10].

## Supplementary Materials

The following are available online: crystallographic data for compound **16**H^+^Cl^−^, **17**H^+^Cl^−^ and **20**H^+^Cl^−^ in crystallographic information file (CIF) format, at https://www.mdpi.com/1420-3049/25/7/1697/s1. The data were submitted to the Cambridge Structural Database and given the following CCDC deposition numbers 1980942–1980944. These data can be obtained free of charge via http://www.ccdc.cam.ac.uk/conts/retrieving.html.

## References

[1] Ferguson F.M., Gray N.S. (2018). Kinase inhibitors: The road ahead. Nat. Rev. Drug Discov..

[2] Cohen P., Alessi D.R. (2013). Kinase drug discovery—What’s next in the field?. ACS Chem. Biol..

[3] Manning G., Whyte D.B., Martinez R., Hunter T., Sudarsanam S. (2002). The Protein Kinase Complement of the Human Genome. Science.

[4] Knapp S., Arruda P., Blagg J., Burley S., Drewry D.H., Edwards A., Fabbro D., Gillespie P., Gray N.S., Kuster B. (2013). A public-private partnership to unlock the untargeted kinome. Nat. Chem. Biol..

[5] Vagnozzi R.J., Gatto G.J., Kallander L.S., Hoffman N.E., Mallilankaraman K., Ballard V.L.T., Lawhorn B.G., Stoy P., Philp J., Graves A.P. (2013). Inhibition of the cardiomyocyte-specific TNNI3K limits oxidative stress, injury, and adverse remodeling in the ischemic heart. Sci. Transl. Med..

[6] Wang L., Wang H., Ye J., Xu R.X., Song L., Shi N., Zhang Y.W., Chen X., Meng X.M. (2011). Adenovirus-mediated overexpression of cardiac troponin I-interacting kinase promotes cardiomyocyte hypertrophy. Clin. Exp. Pharmacol. Physiol..

[7] Wheeler F.C., Tang H., Marks O.A., Hadnott T.N., Chu P.L., Mao L., Rockman H.A., Marchuk D.A. (2009). Tnni3k modifies disease progression in murine models of cardiomyopathy. PLoS Genet..

[8] Lawhorn B.G., Philp J., Zhao Y., Louer C., Hammond M., Cheung M., Fries H., Graves A.P., Shewchuk L., Wang L. (2015). Identification of Purines and 7-Deazapurines as Potent and Selective Type I Inhibitors of Troponin I-Interacting Kinase (TNNI3K). J. Med. Chem..

[9] Lawhorn B.G., Philp J., Graves A.P., Holt D.A., Gatto G.J., Kallander L.S. (2016). Substituent Effects on Drug-Receptor H-bond Interactions: Correlations Useful for the Design of Kinase Inhibitors. J. Med. Chem..

[10] Lawhorn B.G., Philp J., Graves A.P., Shewchuk L., Holt D.A., Gatto G.J., Kallander L.S. (2016). GSK114: A selective inhibitor for elucidating the biological role of TNNI3K. Bioorg Med. Chem Lett..

[11] Philp J., Lawhorn B.G., Graves A.P., Shewchuk L., Rivera K.L., Jolivette L.J., Holt D.A., Gatto G.J., Kallander L.S. (2018). 4,6-Diaminopyrimidines as Highly Preferred Troponin I-Interacting Kinase (TNNI3K) Inhibitors. J. Med. Chem..

[12] Asquith C.R.M., Laitinen T., Bennett J.M., Godoi P.H., East M.P., Tizzard G.J., Graves L.M., Johnson G.L., Dornsife R.E., Wells C.I. (2018). Identification and Optimization of 4-Anilinoquinolines as Inhibitors of Cyclin G Associated Kinase. Chem. Med. Chem..

[13] Asquith C.R.M., Berger B.T., Wan J., Bennett J.M., Capuzzi S.J., Crona D.J., Drewry D.H., East M.P., Elkins J.M., Fedorov O. (2019). SGC-GAK-1: A Chemical Probe for Cyclin G Associated Kinase (GAK). J. Med. Chem..

[14] Asquith C.R.M., Treiber D.K., Zuercher W.J. (2019). Utilizing comprehensive and mini-kinome panels to optimize the selectivity of quinoline inhibitors for cyclin G associated kinase (GAK). Bioorg. Med. Chem. Lett..

[15] Asquith C.R.M., Naegeli N., East M.P., Laitinen T., Havener T.M., Wells C.I., Johnson G.L., Drewry D.H., Zuercher W.J., Morris D.C. (2019). Design of a cyclin G associated kinase (GAK)/epidermal growth factor receptor (EGFR) inhibitor set to interrogate the relationship of EGFR and GAK in chordoma. J. Med. Chem..

[16] Asquith C.R.M., Fleck N., Torrice C.D., Crona D.J., Grundner C., Zuercher W.J. (2019). Anti-tubercular activity of novel 4-anilinoquinolines and 4-anilinoquinazolines. Bioorg. Med. Chem. Lett..

[17] Asquith C.R.M., Maffuid K.A., Laitinen T., Torrice C.D., Tizzard G.J., Crona D.J., Zuercher W.J. (2019). Targeting an EGFR water network using novel 4-anilinoquin(az)olines inhibitors for chordoma. ChemMedChem.

[18] Asquith C.R.M., Bennett J.M., Su L., Laitinen T., Elkins J.M., Pickett J.E., Wells C.I., Li Z., Willson T.M., Zuercher W.J. (2019). Development of SGC-GAK-1 as an orally active in vivo probe for cyclin G associated kinase through cytochrome P450 inhibition. Molecules.

[19] Asquith C.R.M., Tizzard G.J. (2019). 6-Bromo-*N*-(2-methyl-2*H*-benzo[*d*][1,2,3]triazol-5-yl)quinolin-4-amine. Molbank.

[20] Krimmer S.G., Betz M., Heine A., Klebe G. (2014). Methyl, ethyl, propyl, butyl: Futile but not for water, as the correlation of structure and thermodynamic signature shows in a congeneric series of thermolysin inhibitors. Chem. Med. Chem..

[21] Sheldrick G.M. (2015). ShelXT-Integrated space-group and crystal-structure determination. Acta Crystallogr..

[22] Dolomanov O.V., Bourhis L.J., Gildea R.J., Howard J.A.K., Puschmann H. (2009). Olex2: A complete structure solution, refinement and analysis program. J. Appl. Cryst..

[23] Sheldrick G.M. (2015). Crystal structure refinement with ShelXL. Acta Crystallogr..

[24] Fabian M.A., Biggs W.H., Treiber D.K., Atteridge C.E., Azimioara M.D., Benedetti M.G., Carter T.A., Ciceri P., Edeen P.T., Floyd M. (2005). A small molecule-kinase interaction map for clinical kinase inhibitors. Nat. Biotechnol..

