# Supplementary material for: New Insights into 4-Anilinoquinazolines as Inhibitors of Cardiac Troponin I–Interacting Kinase (TNNi3K)
Source: Molecules. 2020 Apr 7;25(7):1697. doi: 10.3390/molecules25071697 (PMC7180948; doi:10.3390/molecules25071697)
Supplement: Supplementary file 1 [file molecules-25-01697-s001.pdf]

1. Characterisation of compounds 2, 7, 12, 13, 15-20
2. LabBook Codes and SMILES for 2, 7, 15-17, 20
3. KINOMEscan® results of 7
4. KINOMEscan® results of 15
5. Mass Spectrometry Method

1. Compound Characterisation

4-(dimethylamino)-*N*-methyl-3-nitrobenzene-1-sulfonamide (**12**)

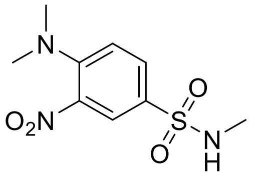

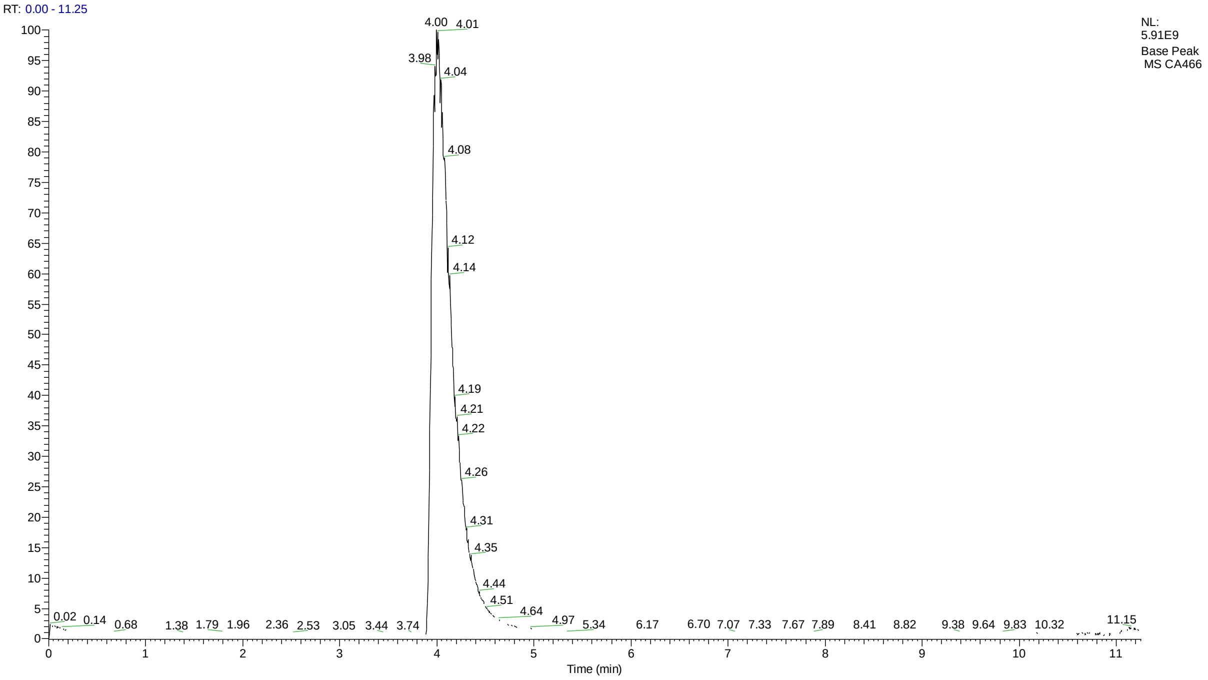

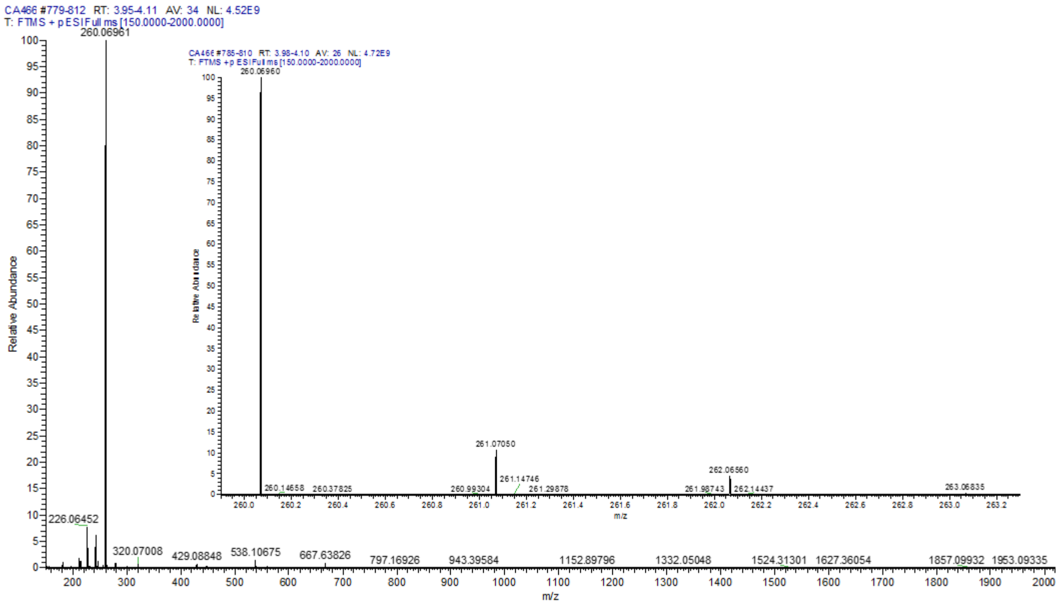

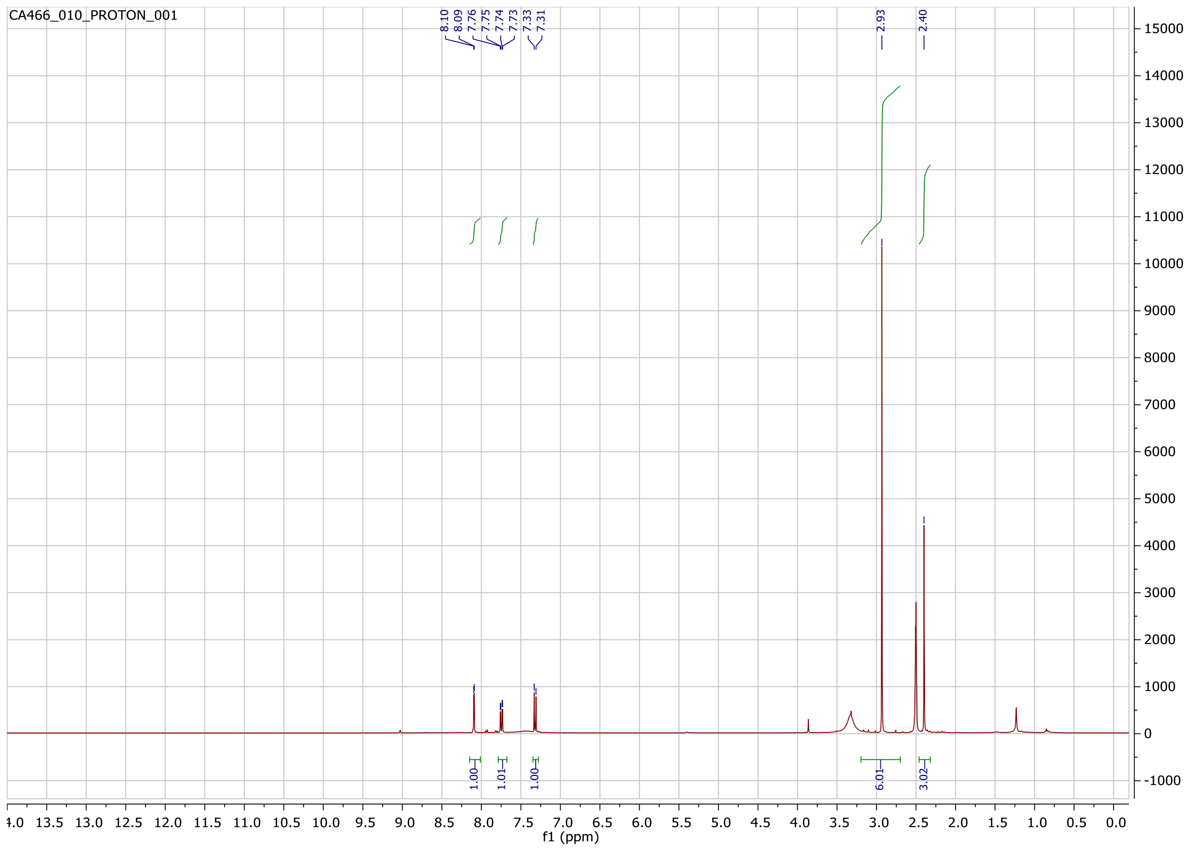

CA466_010_PROTON_001
8.10
8.09
7.76
7.75
7.74
7.73
7.33
7.31
2.93
2.40
1.00
1.01
1.00
6.01
3.02
f1 (ppm)

3-amino-4-(dimethylamino)-*N*-methylbenzene-1-sulfonamide (**13**)

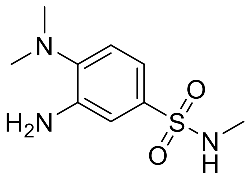

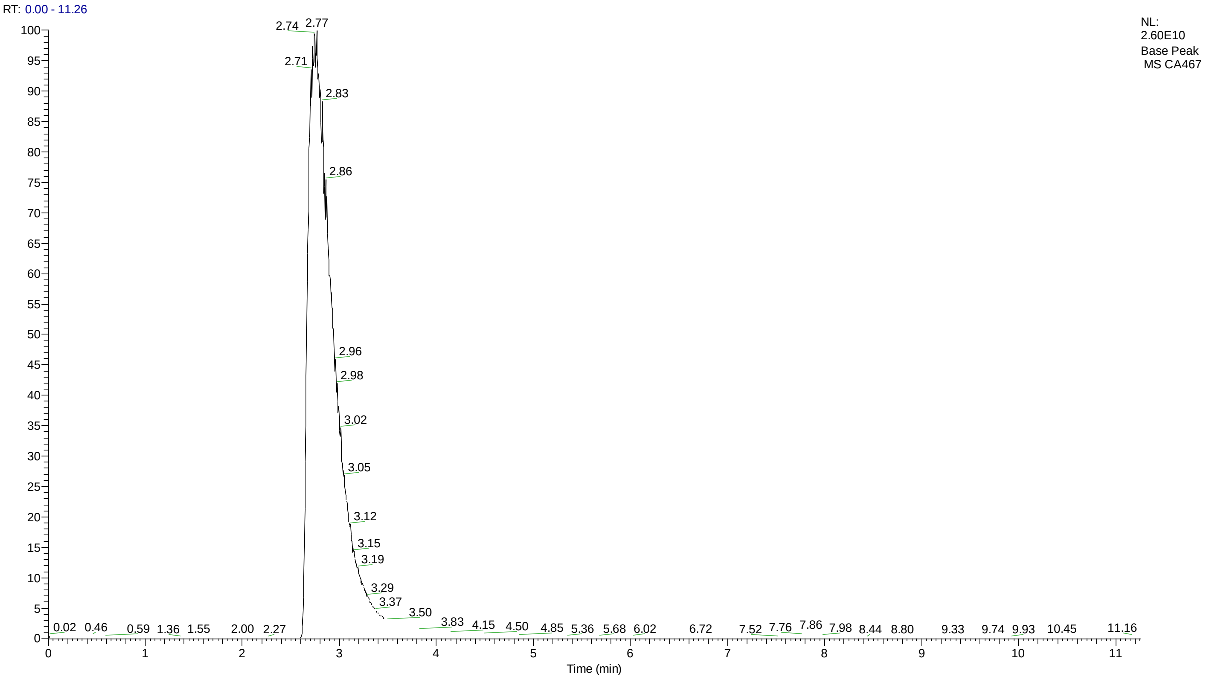

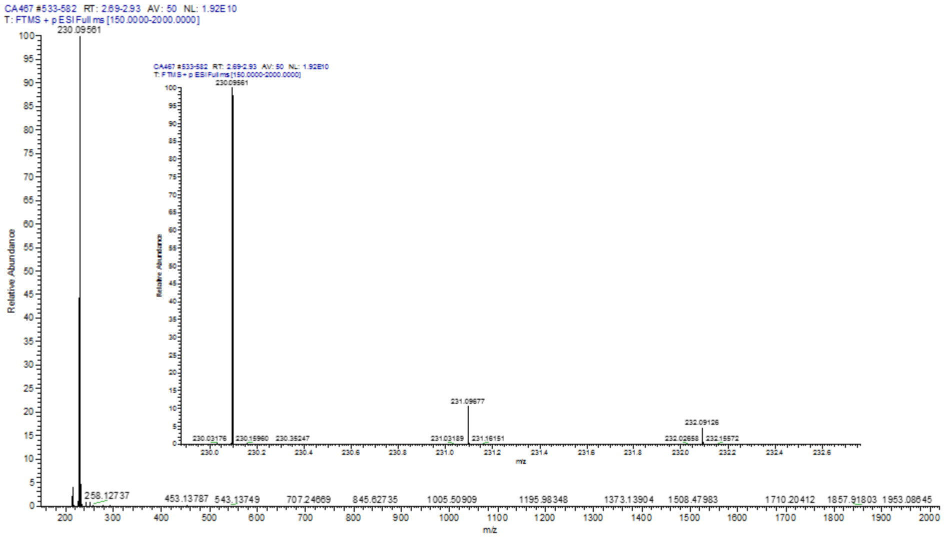

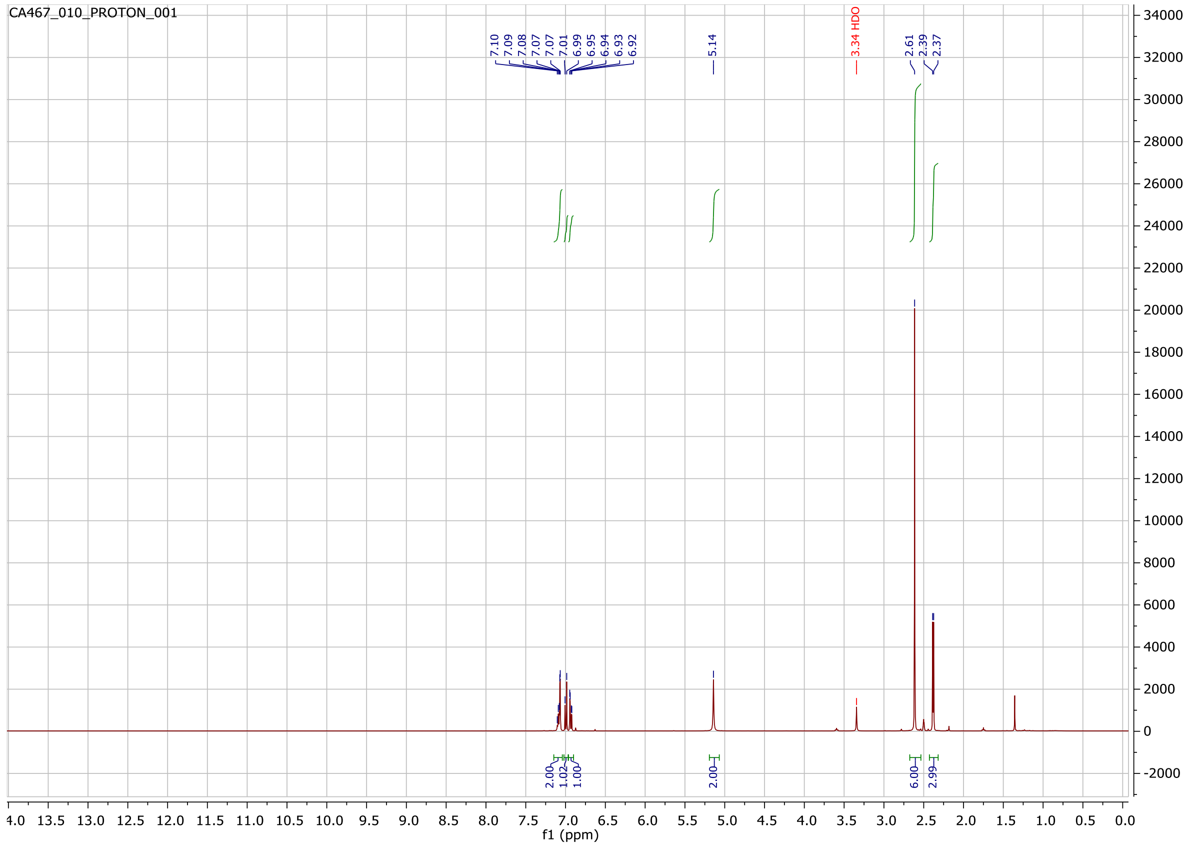
CA467_010_PROTON_001
7.10
7.09
7.08
7.07
7.07
7.01
6.99
6.95
6.94
6.93
6.92
5.14
3.34 HDO
2.61
2.39
2.37
2.00
1.02
1.00
2.00
6.00
2.99
f1 (ppm)

3-((6,7-dimethoxyquinazolin-4-yl)amino)-4-(dimethylamino)-*N*-methylbenzenesulfonamide (**7**)

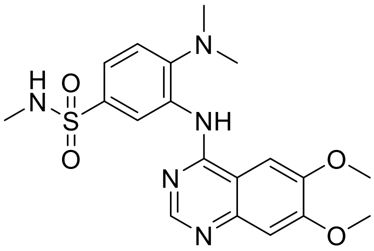

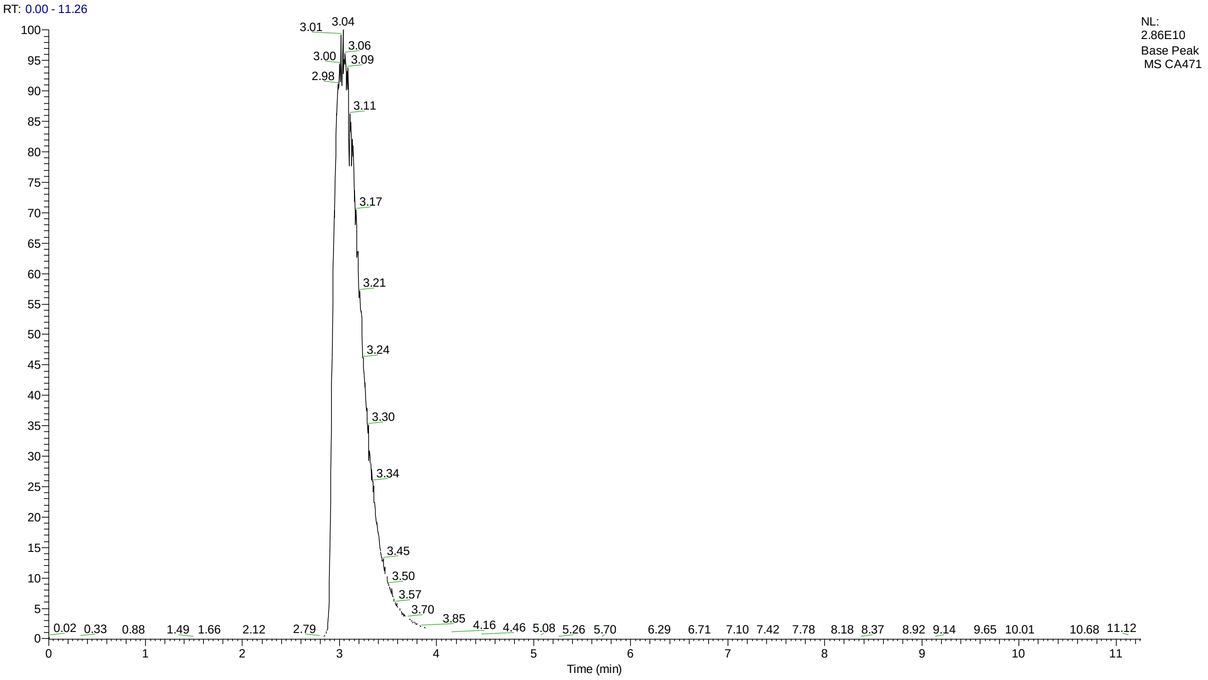

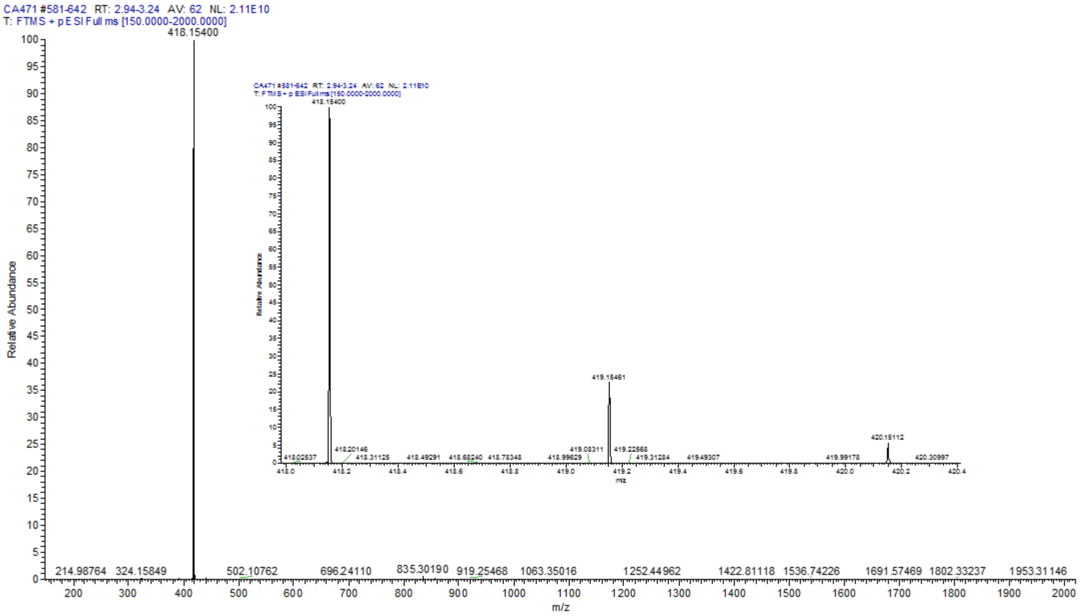

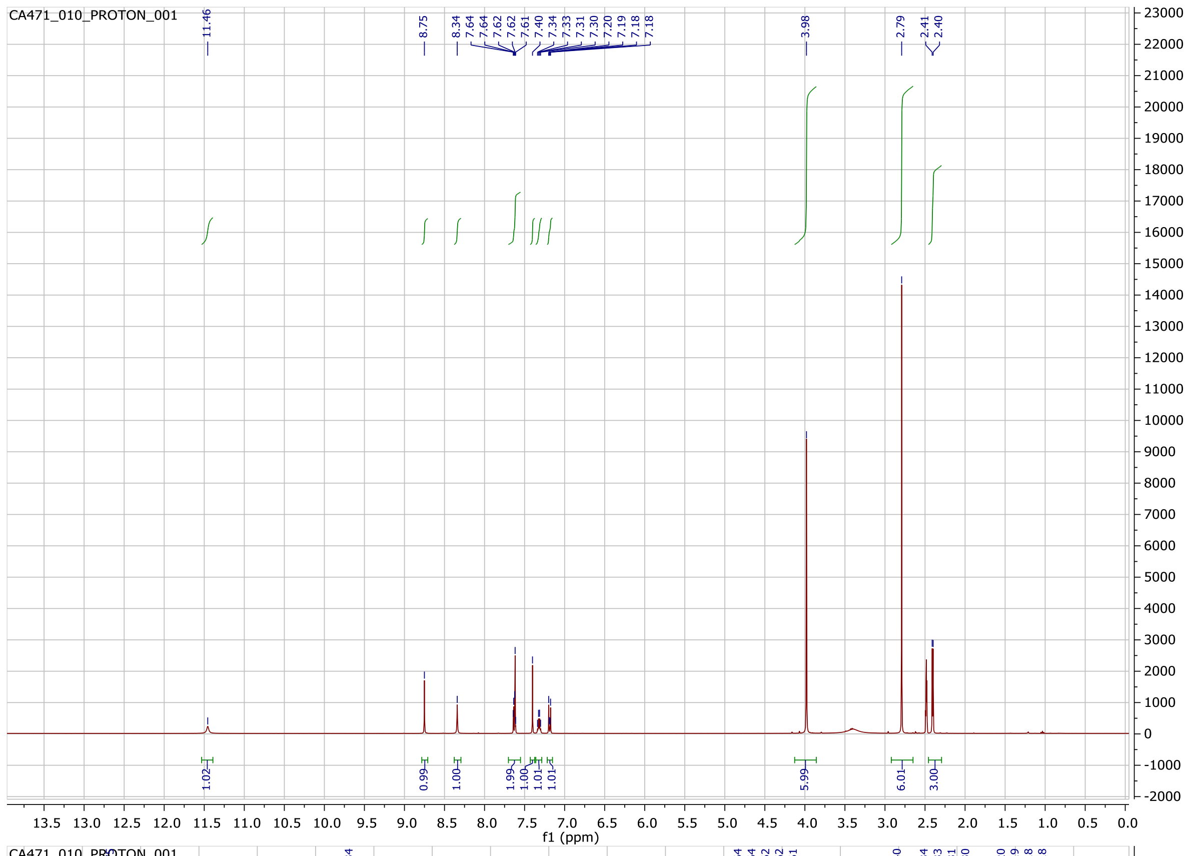
CA471_010_PROTON_001
11.46
8.75
8.34
7.64
7.64
7.62
7.62
7.61
7.40
7.34
7.33
7.31
7.30
7.20
7.19
7.18
7.18
3.98
2.79
2.41
2.40
1.02
0.99
1.00
1.99
1.00
1.01
1.01
5.99
6.01
3.00
f1 (ppm)

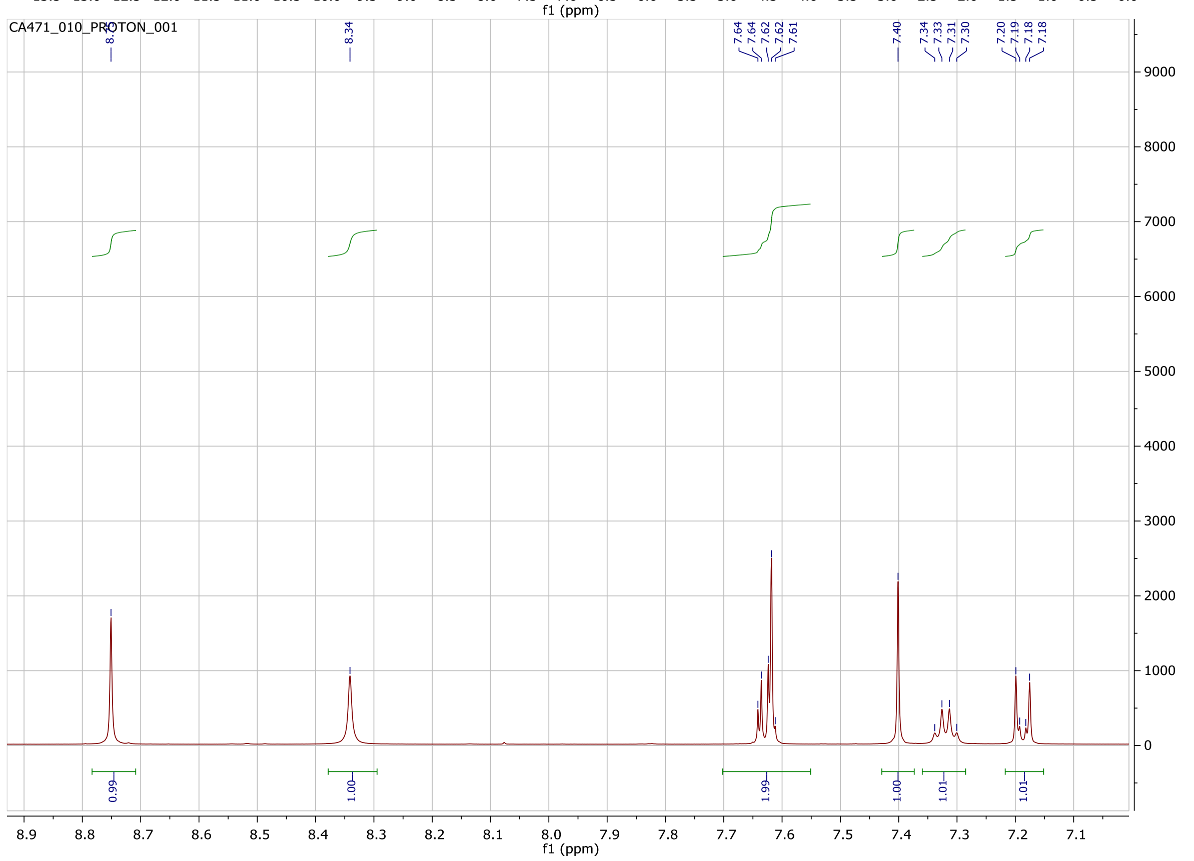
CA471_010_PROTON_001
8.34
7.64
7.64
7.62
7.62
7.61
7.40
7.34
7.33
7.31
7.30
7.20
7.19
7.18
7.18
0.99
1.00
1.99
1.00
1.01
1.01
f1 (ppm)

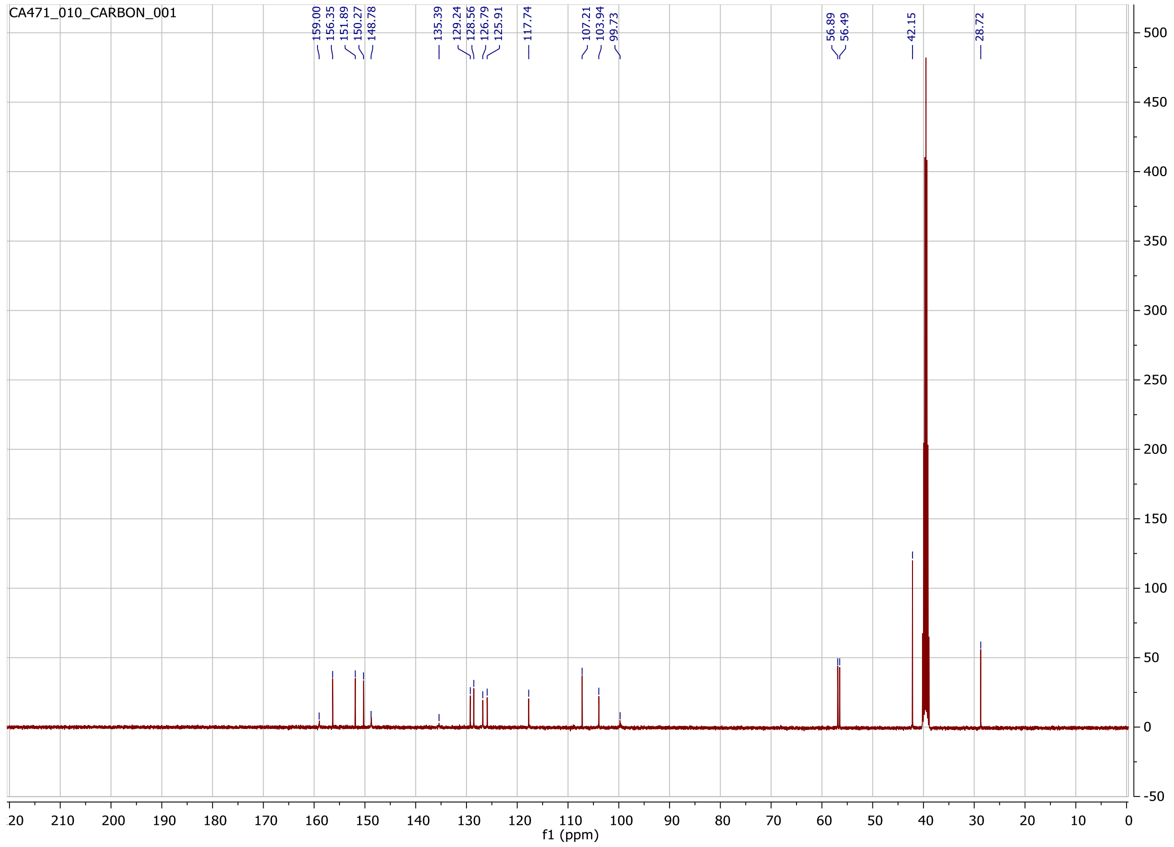
CA471_010_CARBON_001
159.00
156.35
151.89
150.27
148.78
135.39
129.24
128.56
126.79
125.91
117.74
107.21
103.94
99.73
56.89
56.49
42.15
28.72
f1 (ppm)

3-((6,7-dimethoxy-2-methylquinazolin-4-yl)amino)-4-(dimethylamino)-*N*-methylbenzenesulfonamide (**15**)

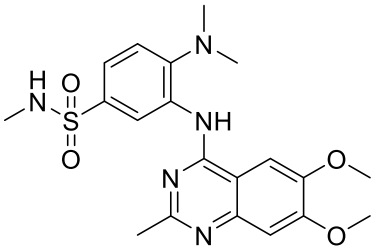

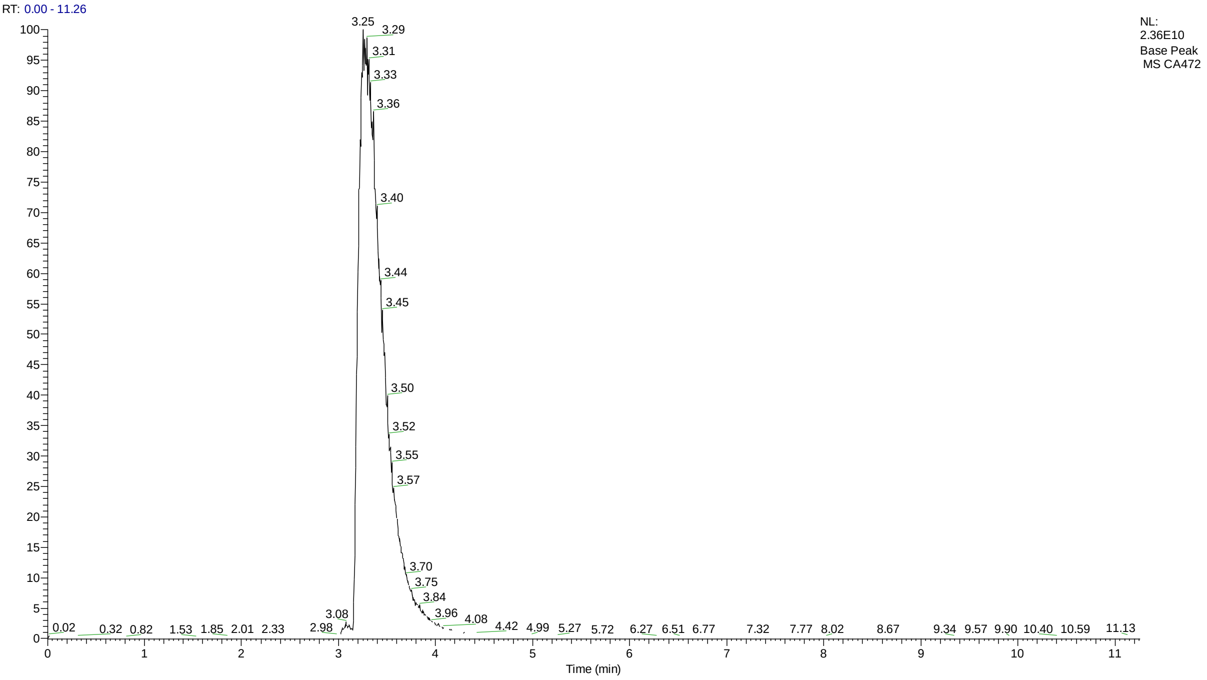

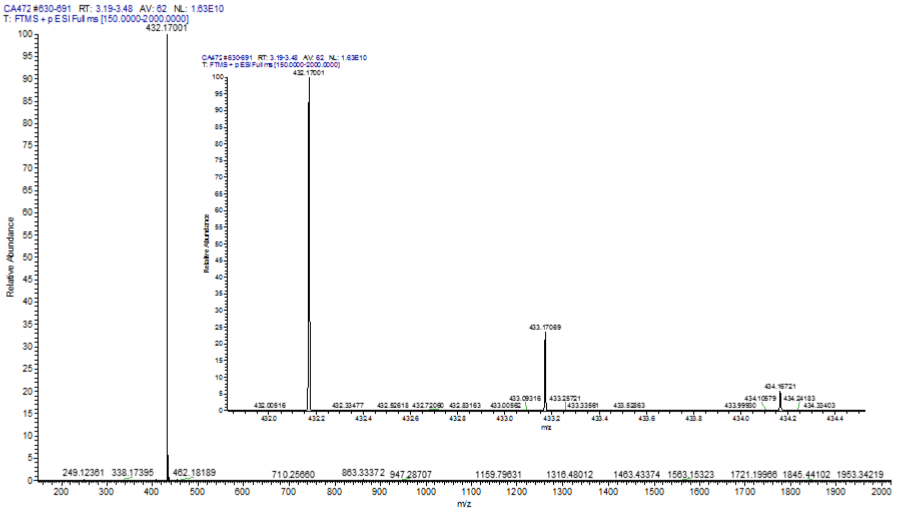

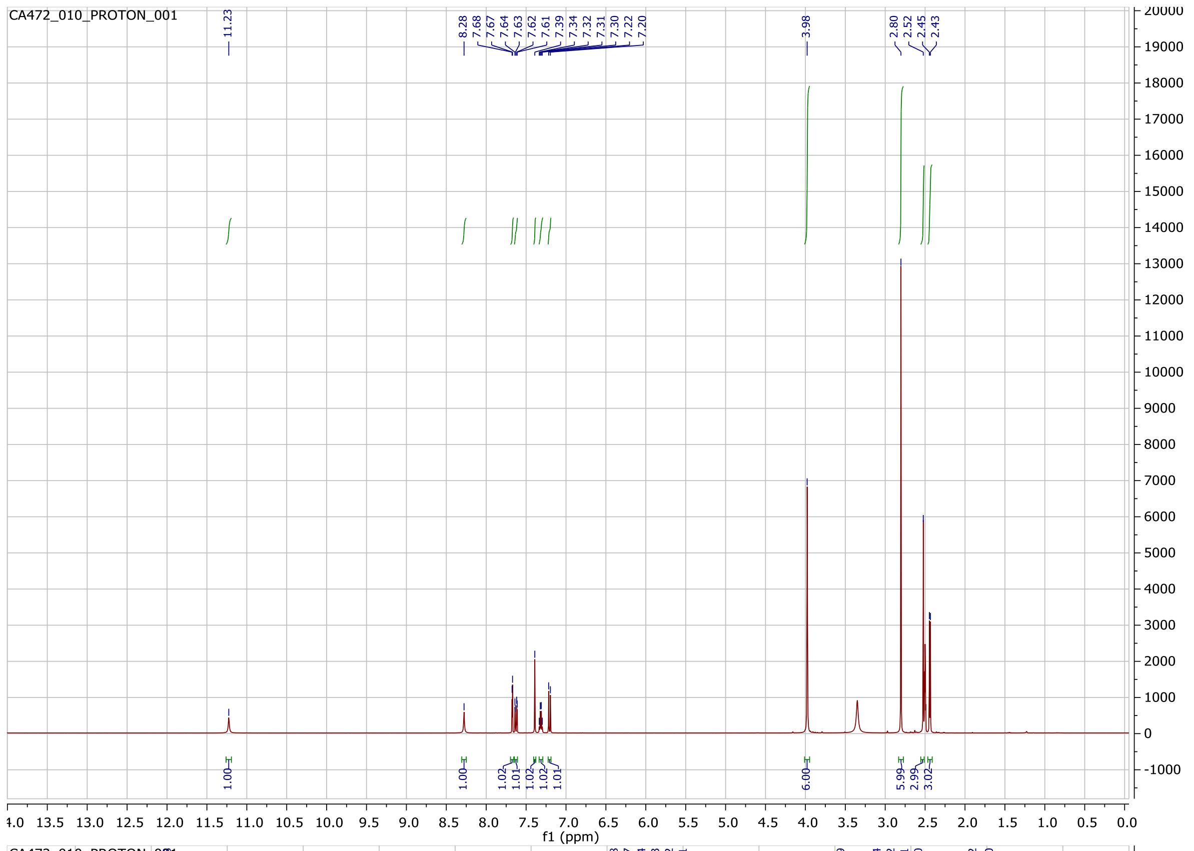
CA472_010_PROTON_001
11.23
8.28
7.68
7.67
7.64
7.63
7.62
7.61
7.39
7.34
7.32
7.31
7.30
7.22
7.20
3.98
2.80
2.52
2.45
2.43
1.00
1.00
1.02
1.01
1.02
1.02
1.01
6.00
5.99
2.99
3.02
f1 (ppm)

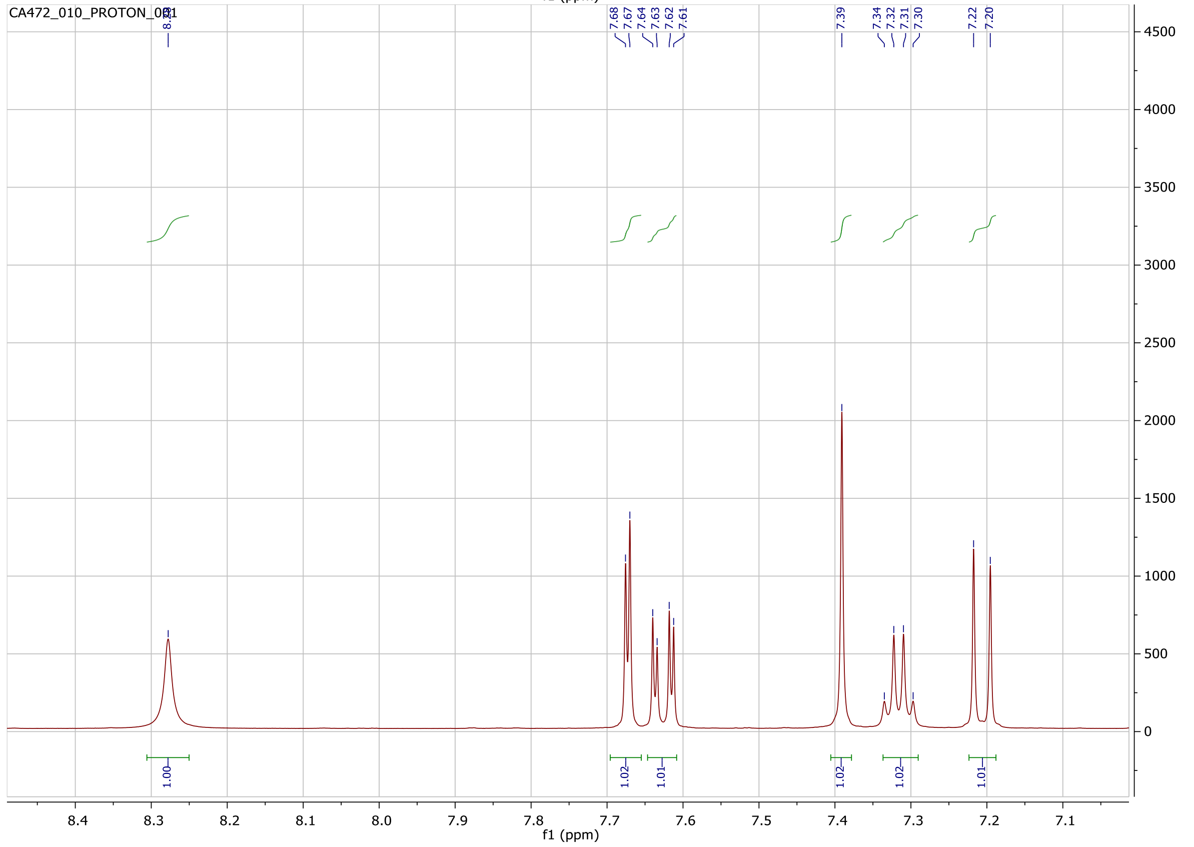
CA472_010_PROTON_001
7.68
7.67
7.64
7.63
7.62
7.61
7.39
7.34
7.32
7.31
7.30
7.22
7.20
1.00
1.02
1.01
1.02
1.02
1.01
f1 (ppm)

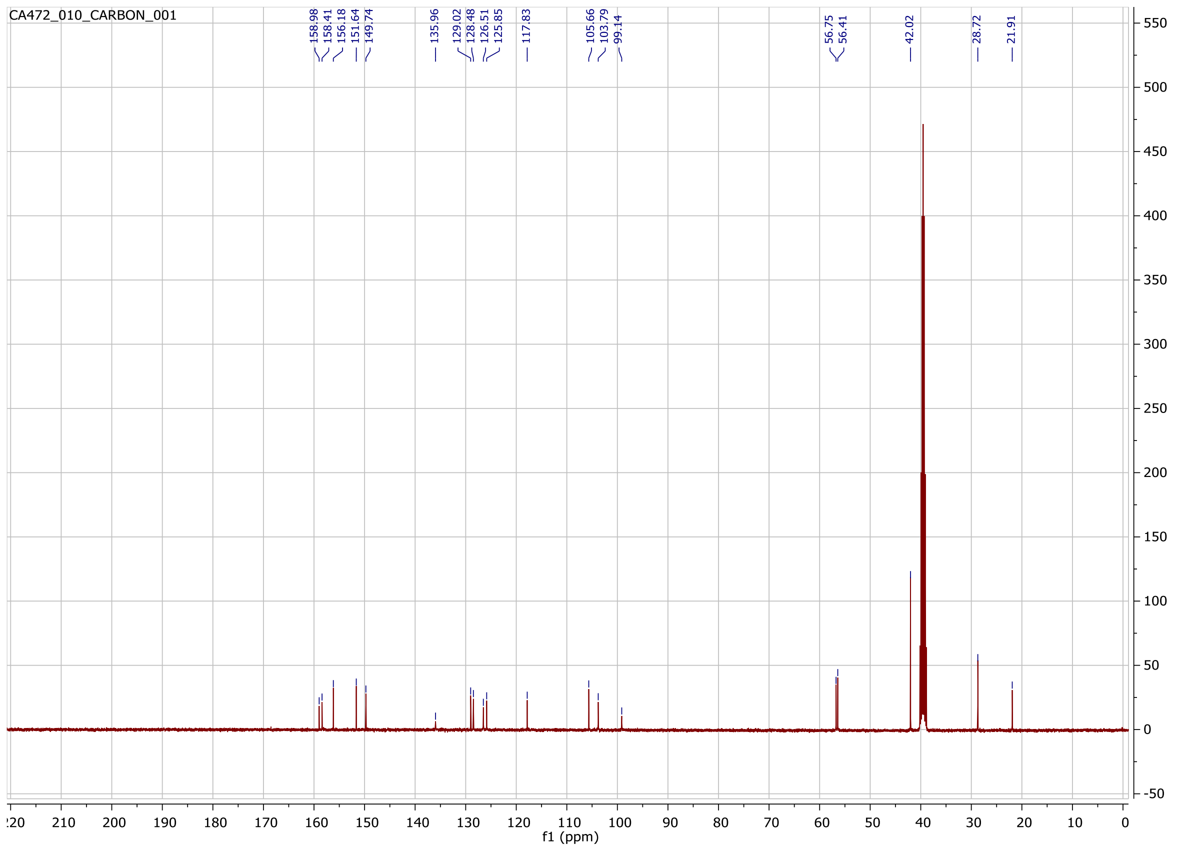

CA472_010_CARBON_001
158.98
158.41
156.18
151.64
149.74
135.96
129.02
128.48
126.51
125.85
117.83
105.66
103.79
99.14
56.75
56.41
42.02
28.72
21.91
f1 (ppm)

4-(dimethylamino)-3-((6-methoxyquinazolin-4-yl)amino)-*N*-methylbenzenesulfonamide (**16**)

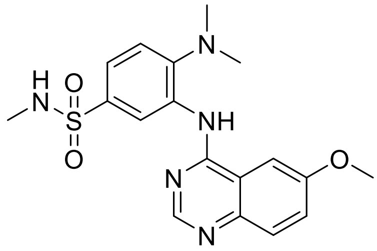

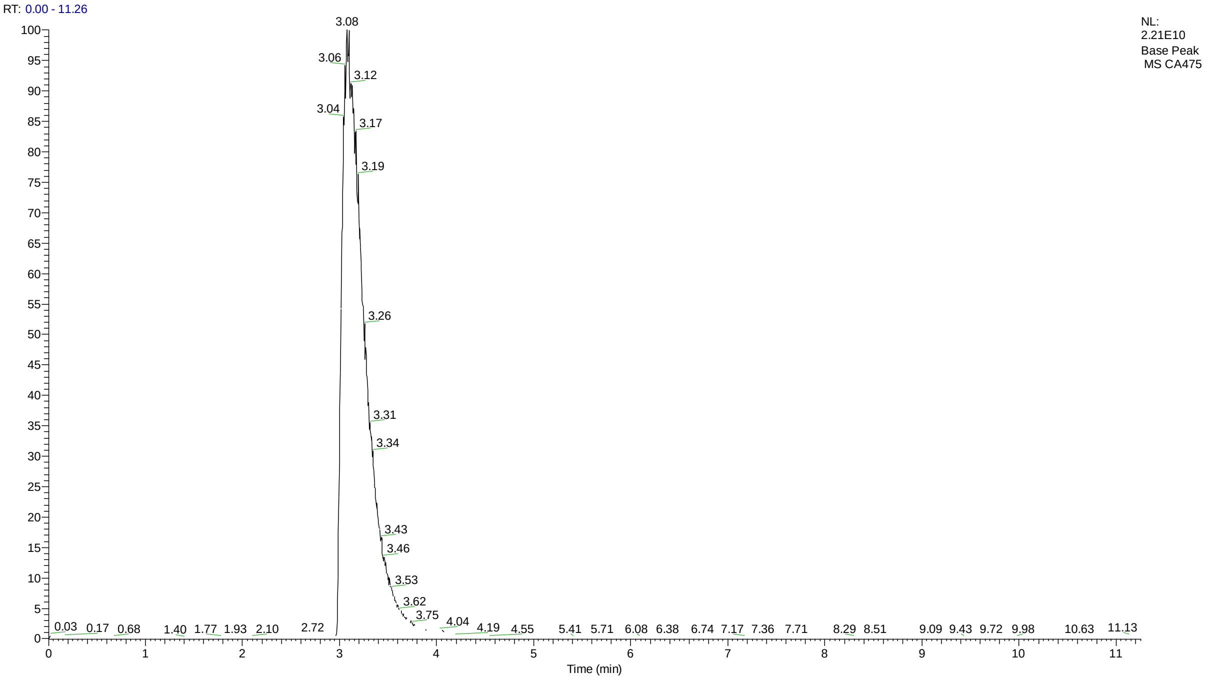

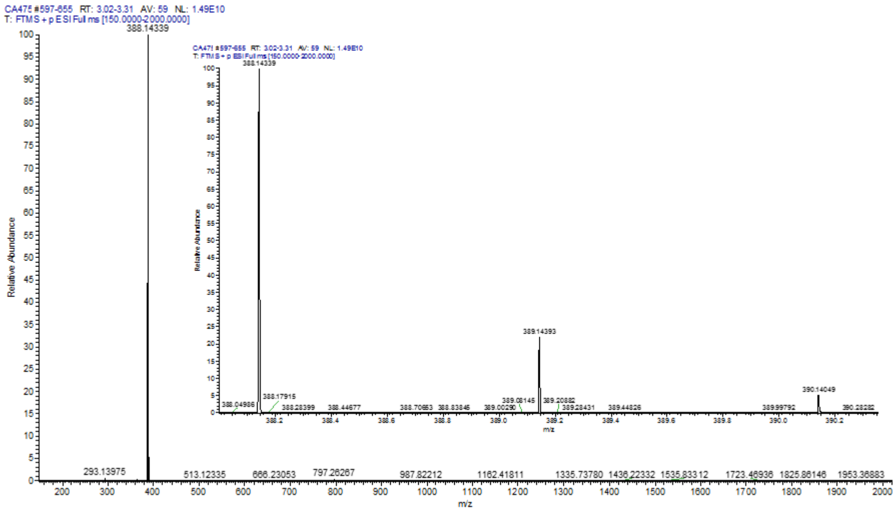

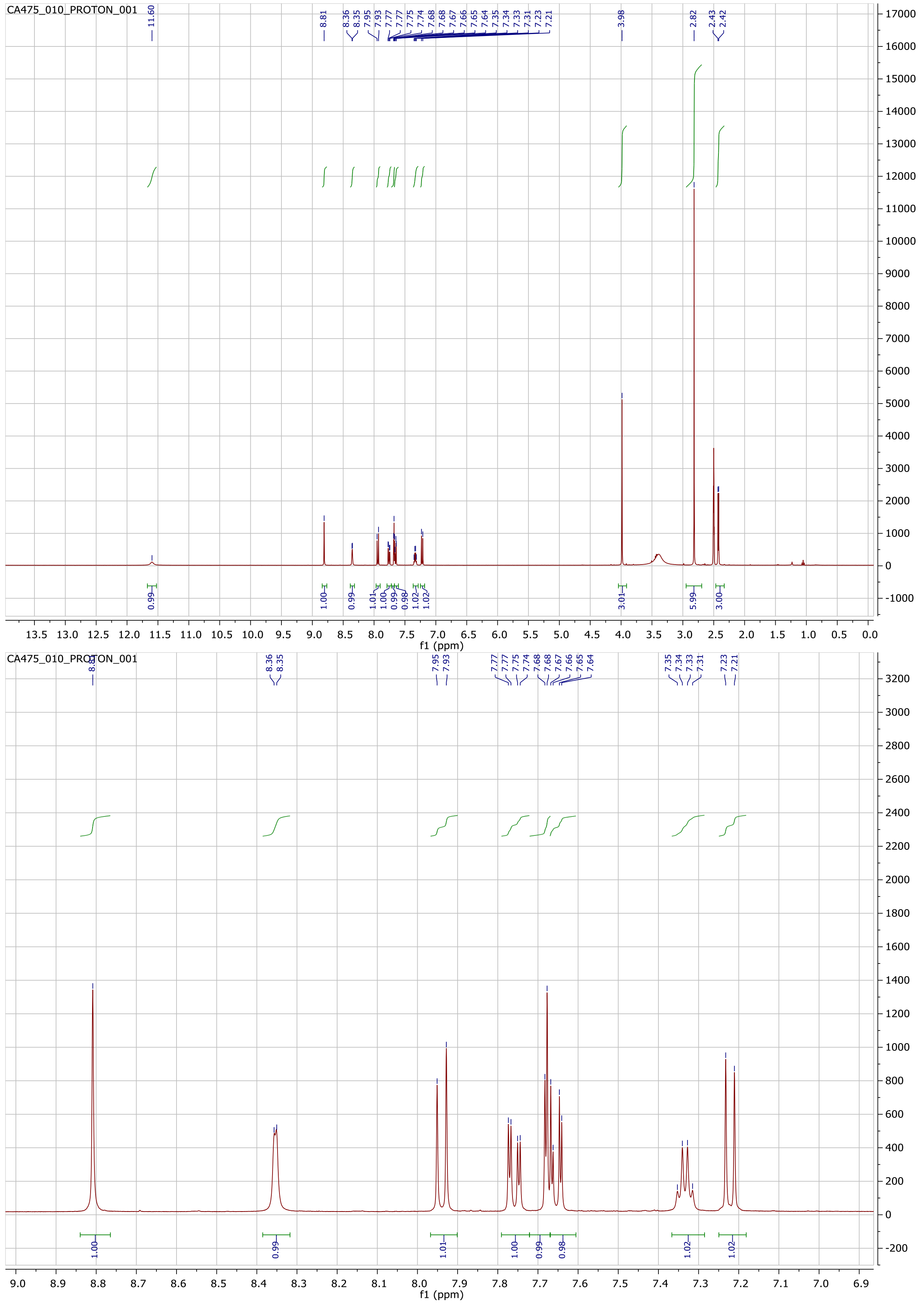
CA475_010_PROTON_001
f1 (ppm)
CA475_010_PROTON_001
f1 (ppm)

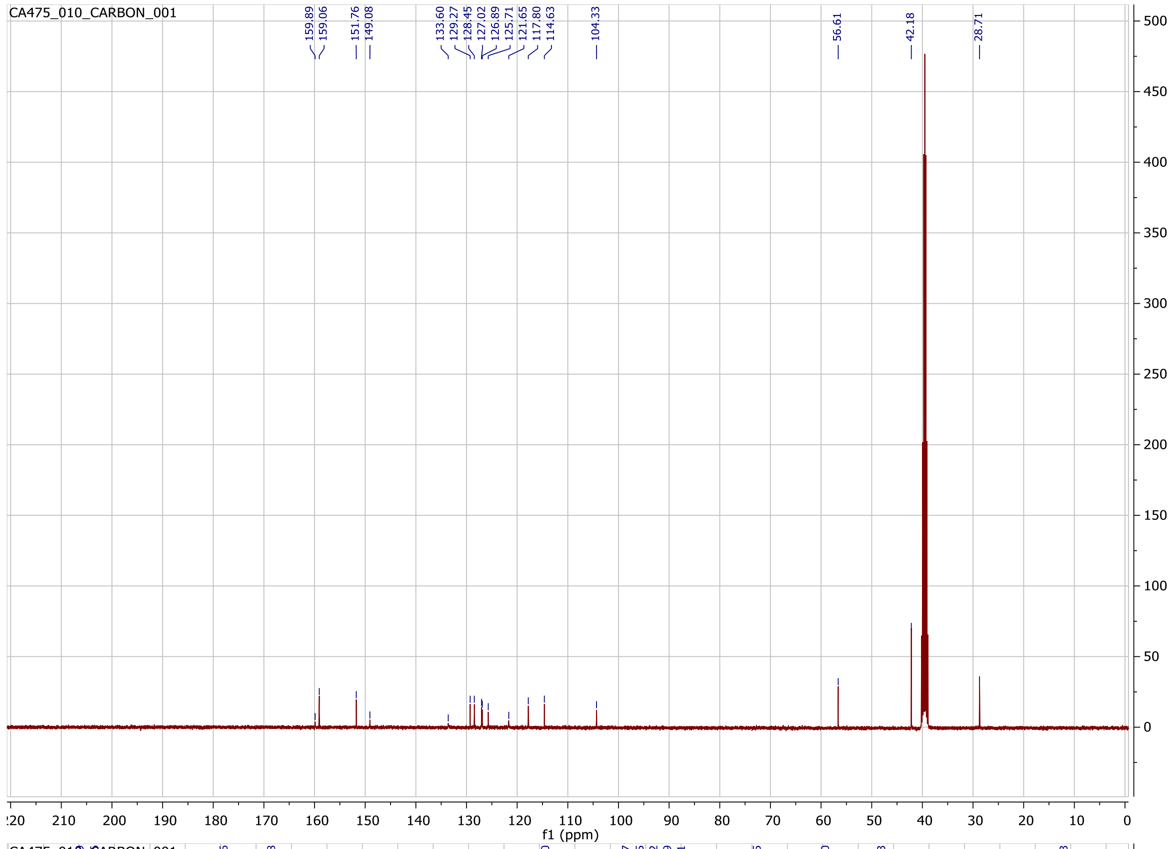
CA475_010_CARBON_001
159.89
159.06
151.76
149.08
133.60
129.27
128.45
127.02
126.89
125.71
121.65
117.80
114.63
104.33
56.61
42.18
28.71
f1 (ppm)

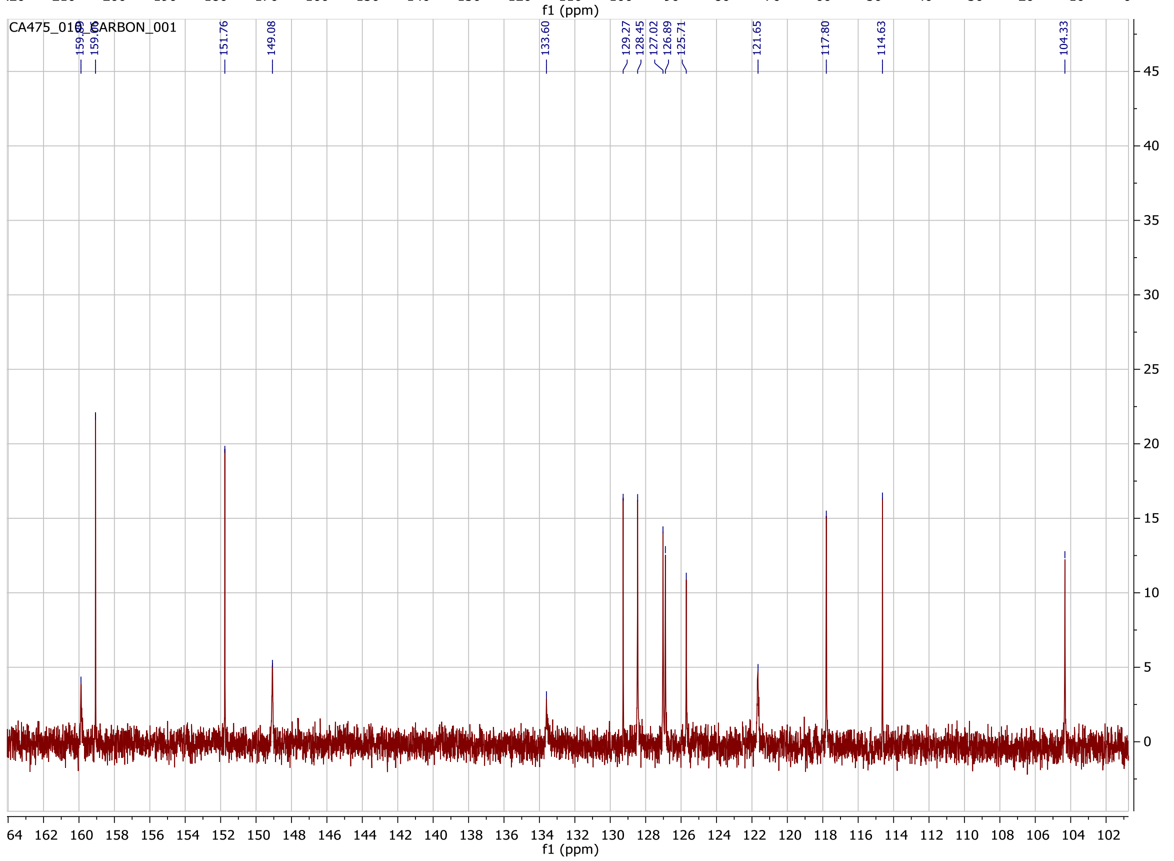
CA475_010_CARBON_001
151.76
149.08
133.60
129.27
128.45
127.02
126.89
125.71
121.65
117.80
114.63
104.33
f1 (ppm)

4-(dimethylamino)-3-((7-methoxyquinazolin-4-yl)amino)-*N*-methylbenzenesulfonamide (**17**)

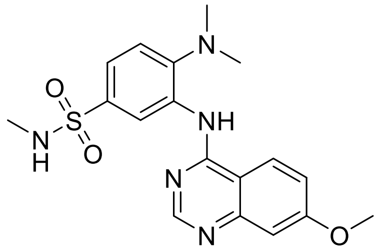

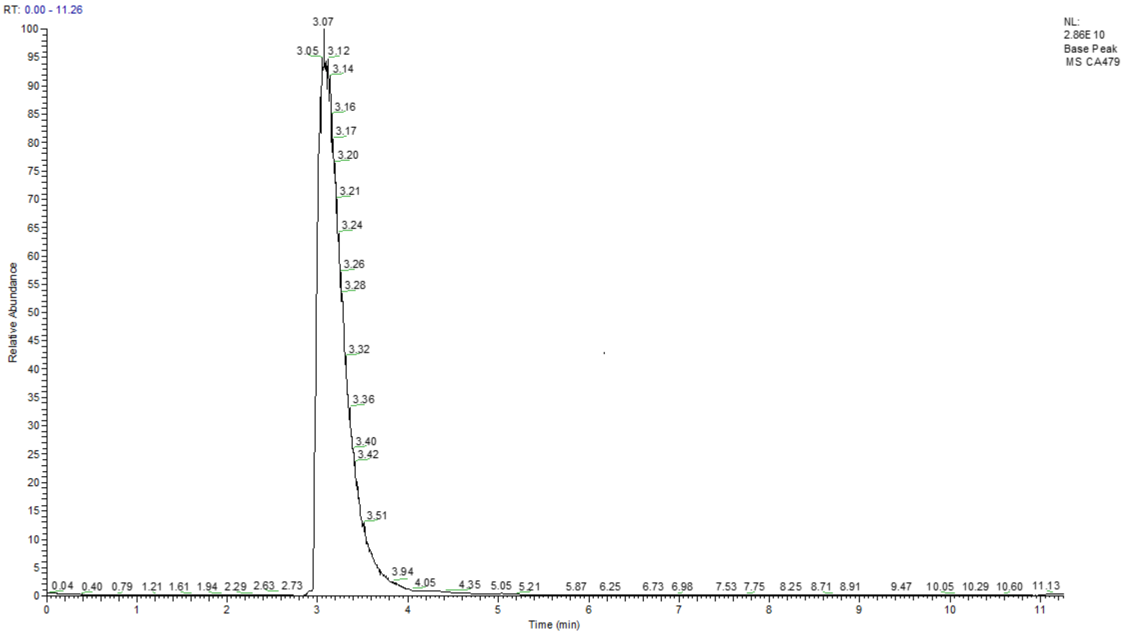

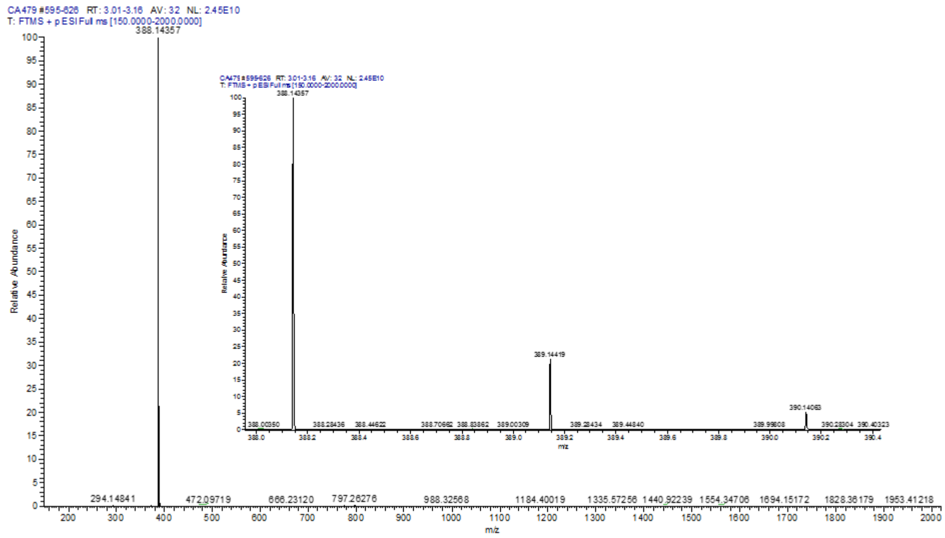

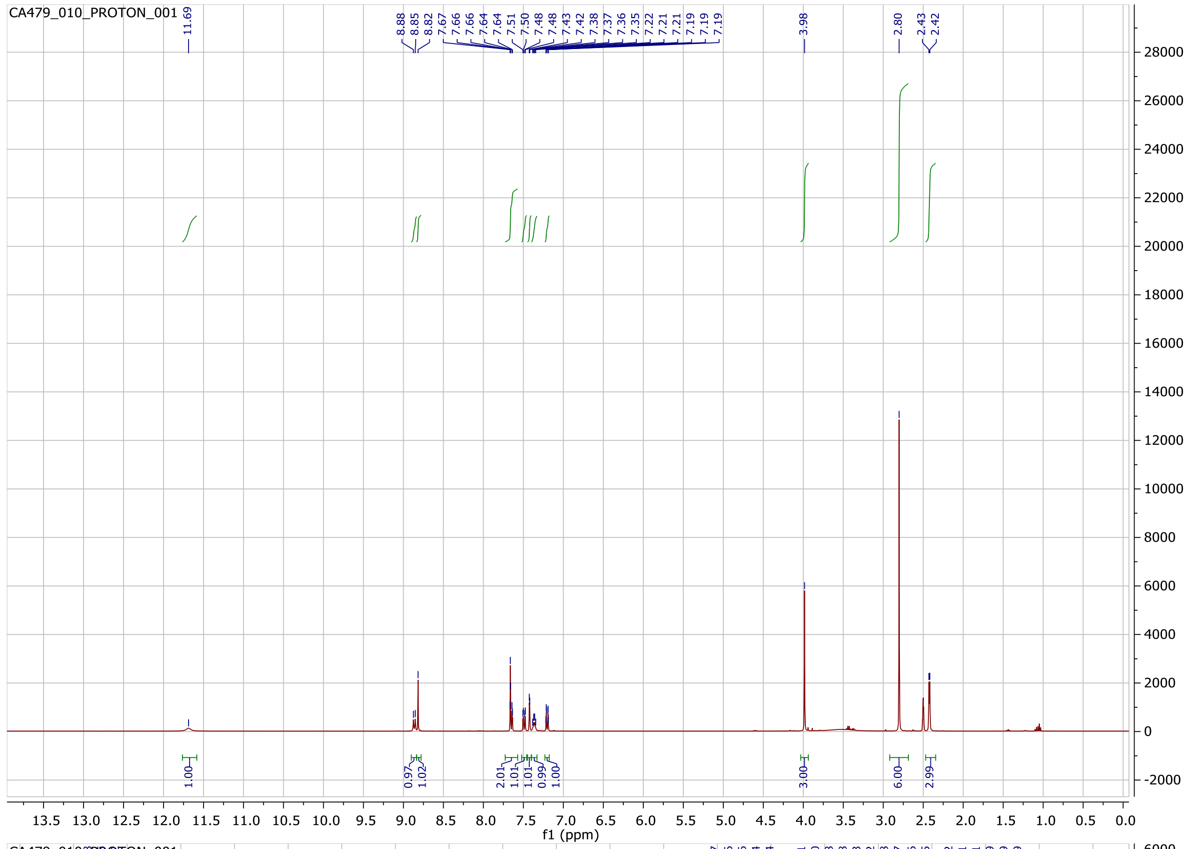
CA479_010_PROTON_001
11.69
8.88
8.85
8.82
7.67
7.66
7.66
7.64
7.51
7.50
7.48
7.48
7.43
7.42
7.38
7.37
7.36
7.35
7.22
7.21
7.21
7.19
7.19
7.19
3.98
2.80
2.43
2.42
1.00
0.97
1.02
2.01
1.01
1.01
0.99
1.00
3.00
6.00
2.99
f1 (ppm)

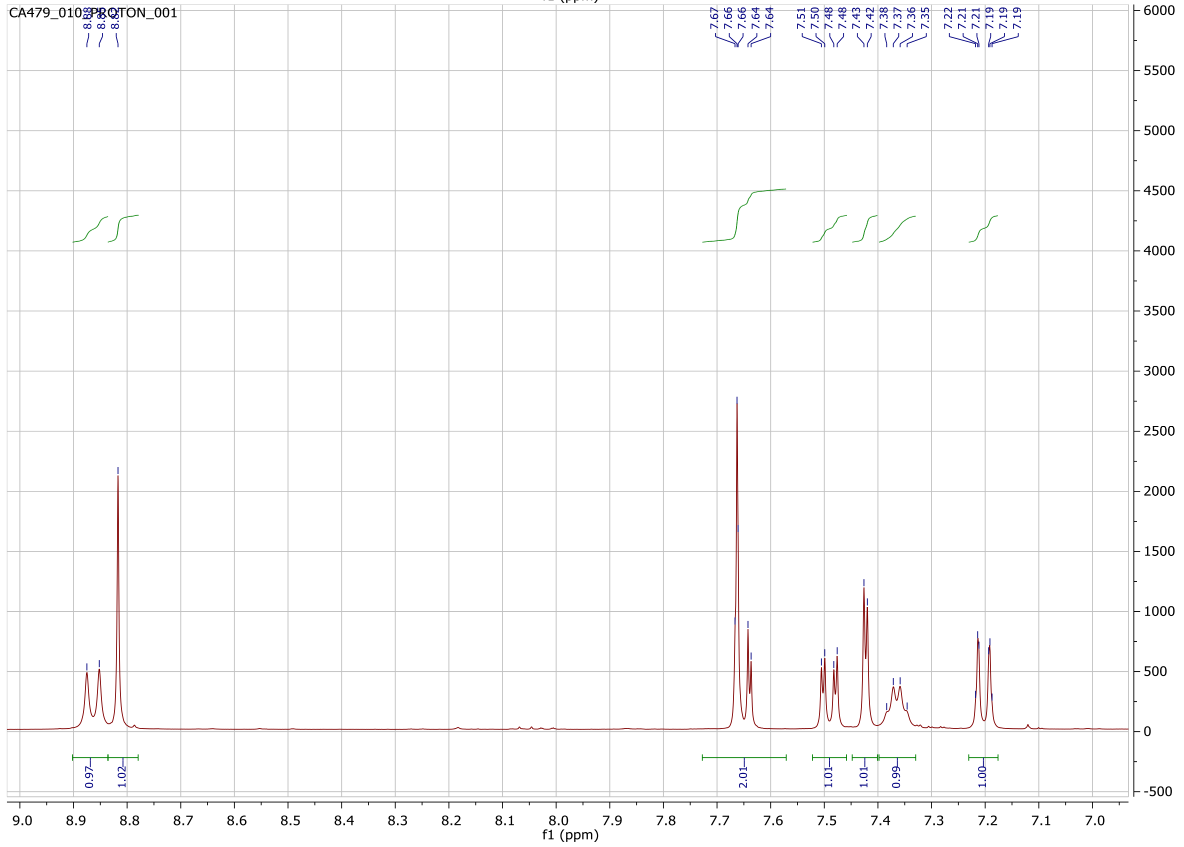
CA479_010_PROTON_001
7.67
7.66
7.66
7.64
7.51
7.50
7.48
7.48
7.43
7.42
7.38
7.37
7.36
7.35
7.22
7.21
7.21
7.19
7.19
7.19
0.97
1.02
2.01
1.01
1.01
0.99
1.00
f1 (ppm)

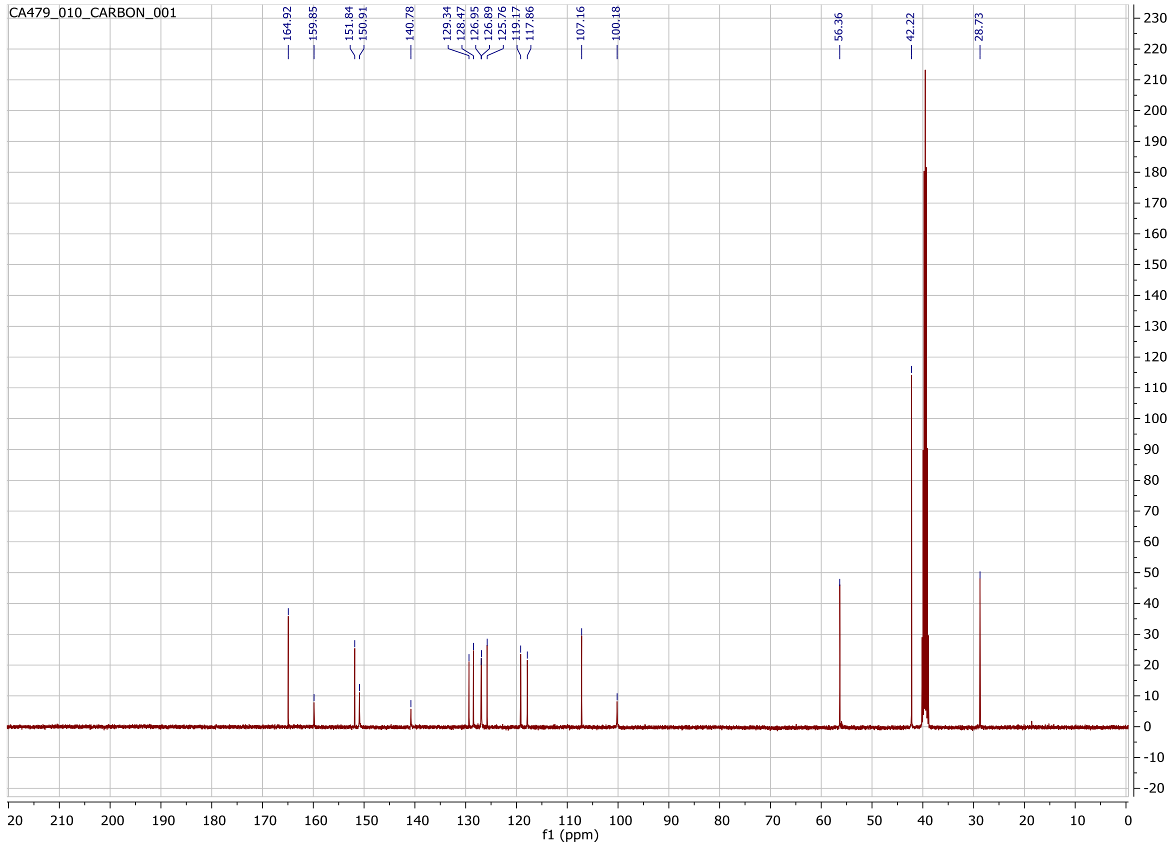

CA479_010_CARBON_001
164.92
159.85
151.84
150.91
140.78
129.34
128.47
126.95
126.89
125.76
119.17
117.86
107.16
100.18
56.36
42.22
28.73
f1 (ppm)

3-((6,7-dimethoxyquinazolin-4-yl)amino)-N-methylbenzenesulfonamide (**2**)

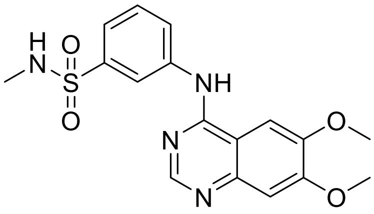

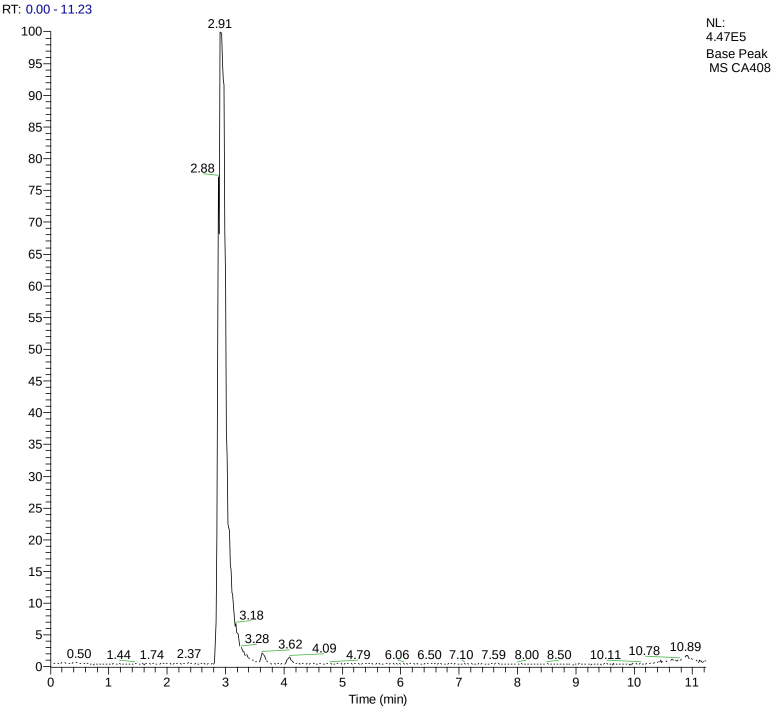

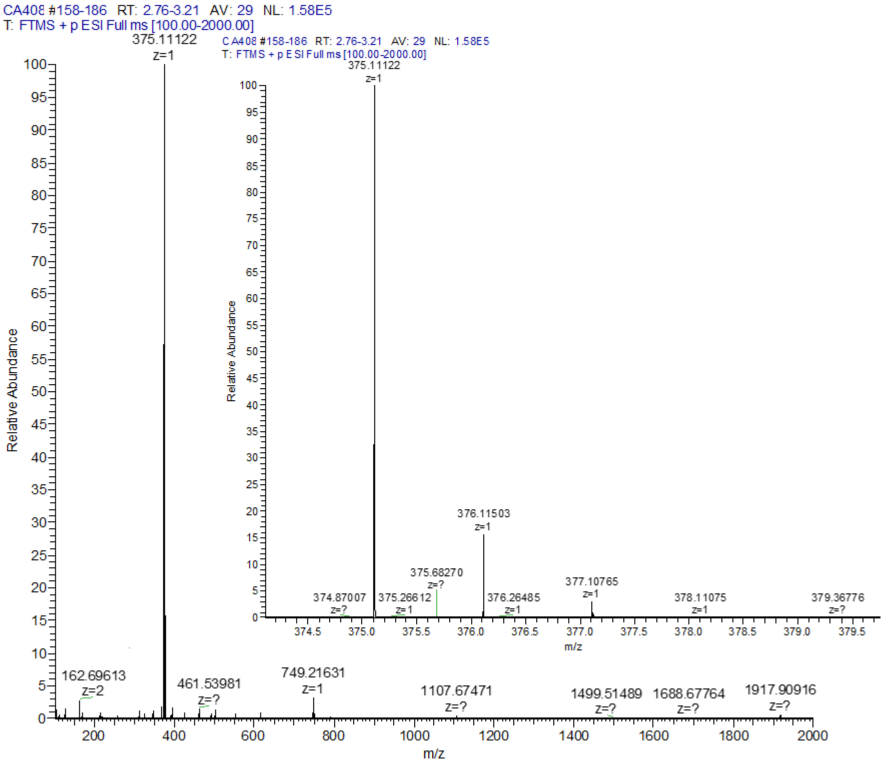

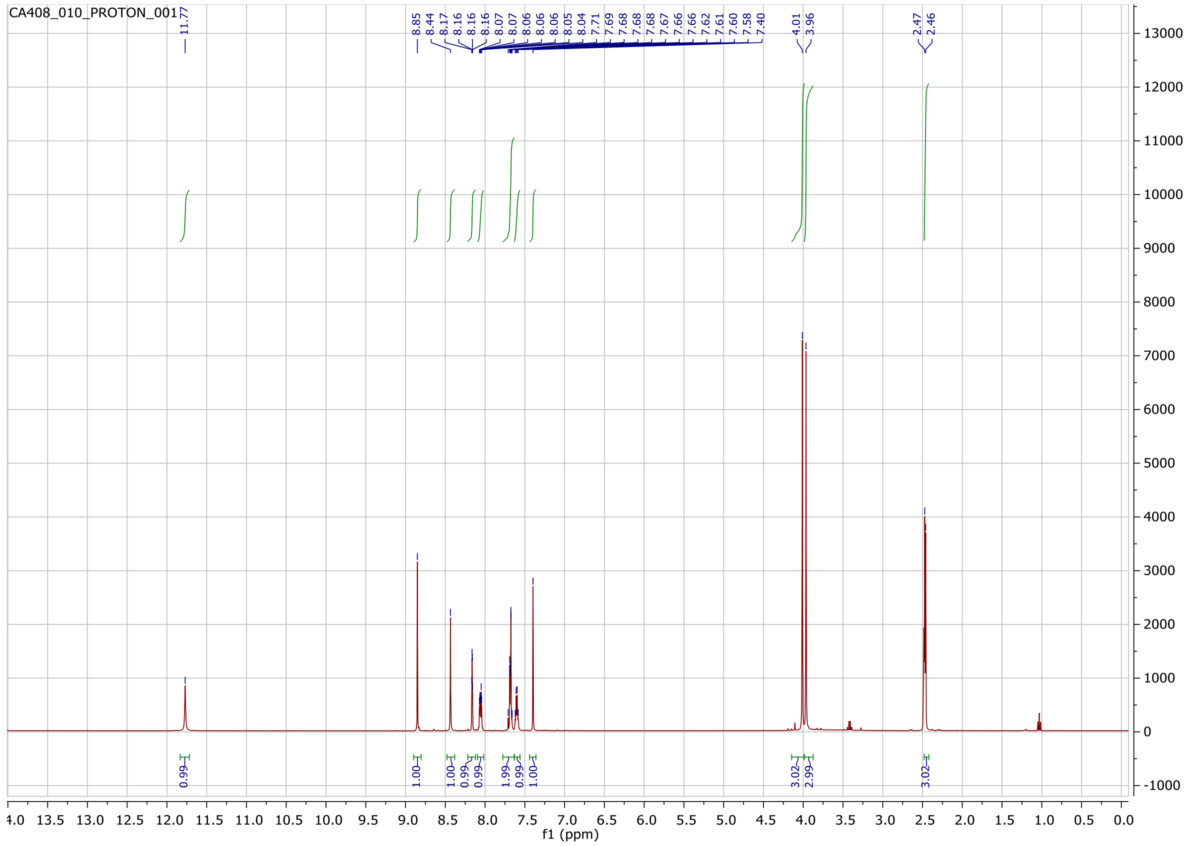
CA408_010_PROTON_001
11.77
8.85
8.44
8.17
8.16
8.16
8.16
8.07
8.06
8.06
8.06
8.05
8.04
7.71
7.69
7.68
7.68
7.67
7.66
7.66
7.62
7.61
7.60
7.58
7.40
4.01
3.96
2.47
2.46
0.99
1.00
1.00
0.99
0.99
1.99
0.99
1.00
3.02
2.99
3.02
f1 (ppm)

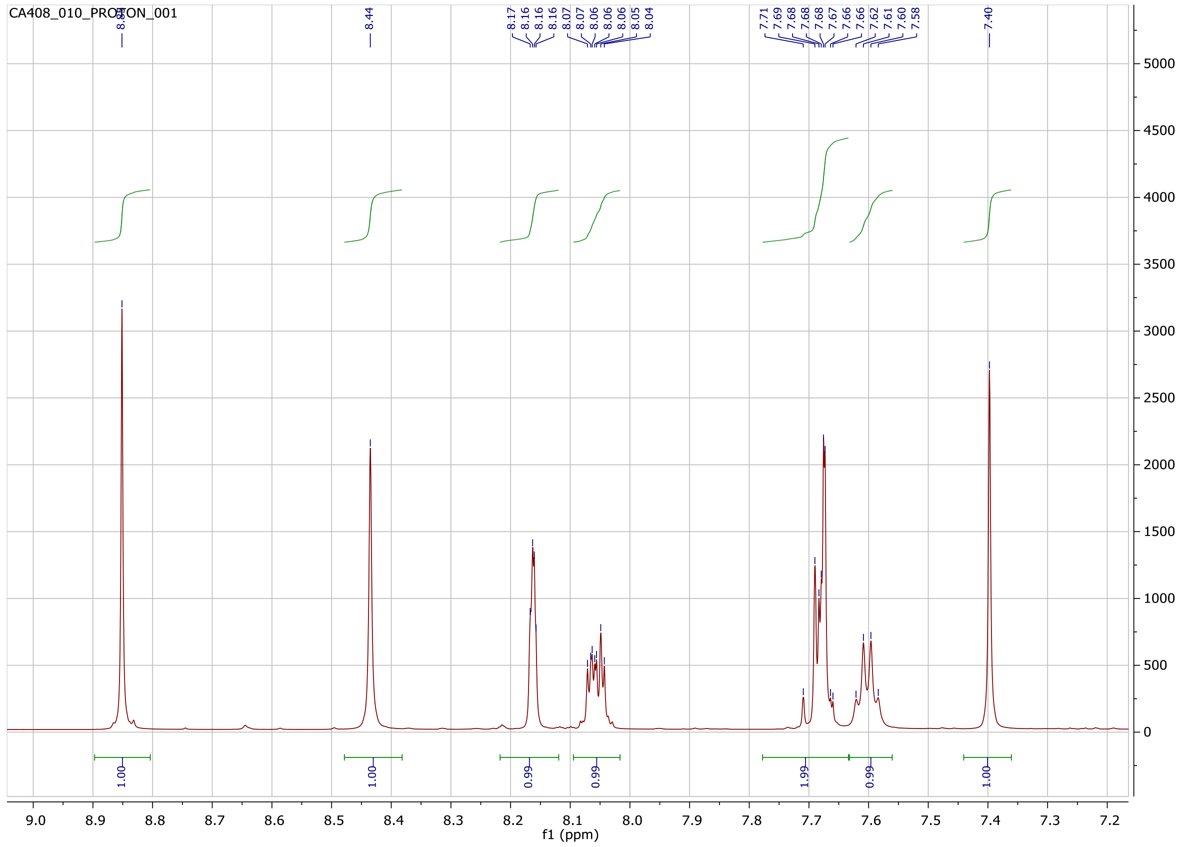
CA408_010_PROTON_001
8.85
8.44
8.17
8.16
8.16
8.16
8.07
8.06
8.06
8.06
8.05
8.04
7.71
7.69
7.68
7.68
7.67
7.66
7.66
7.62
7.61
7.60
7.58
7.40
1.00
1.00
0.99
0.99
1.99
0.99
1.00
f1 (ppm)

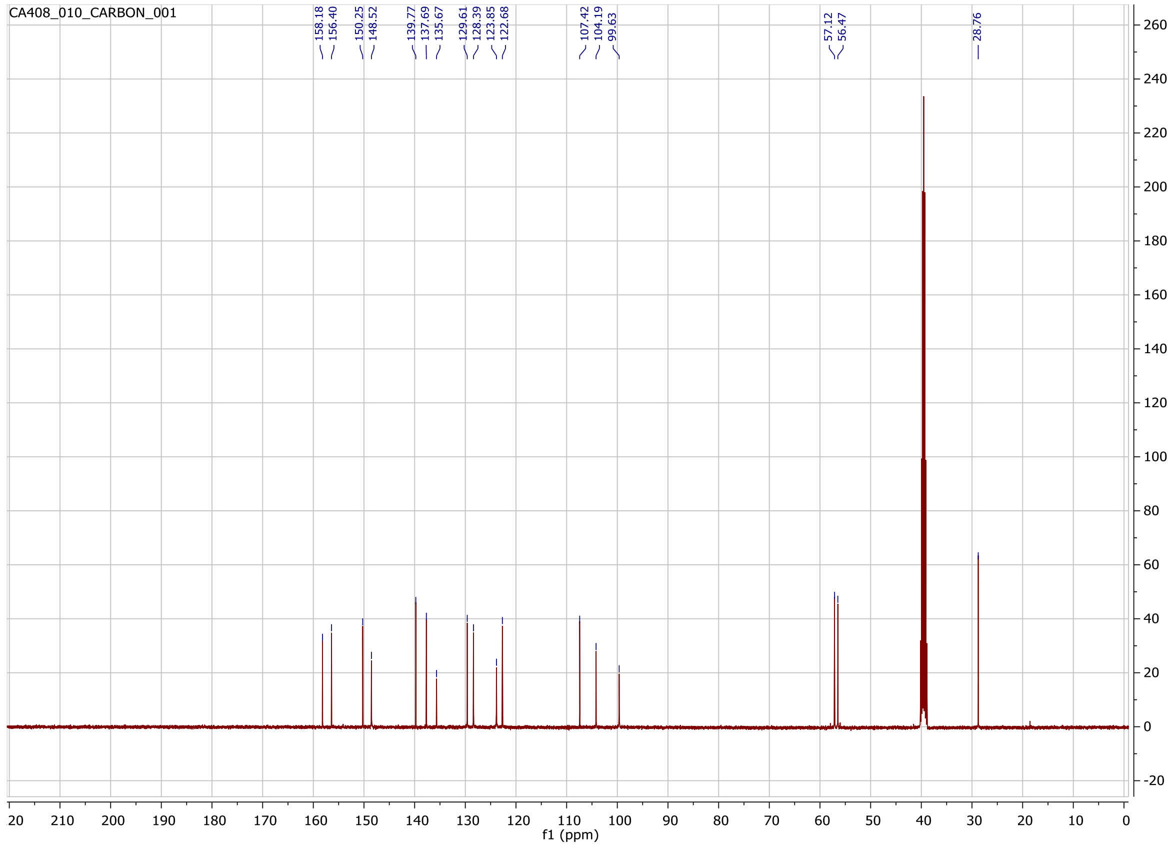
CA408_010_CARBON_001
158.18
156.40
150.25
148.52
139.77
137.69
135.67
129.61
128.39
123.85
122.68
107.42
104.19
99.63
57.12
56.47
28.76
f1 (ppm)

N^1-(6,7-dimethoxyquinazolin-4-yl)-N^2,N^2-dimethylbenzene-1,2-diamine (**20**)

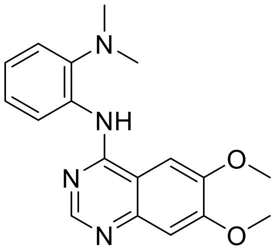

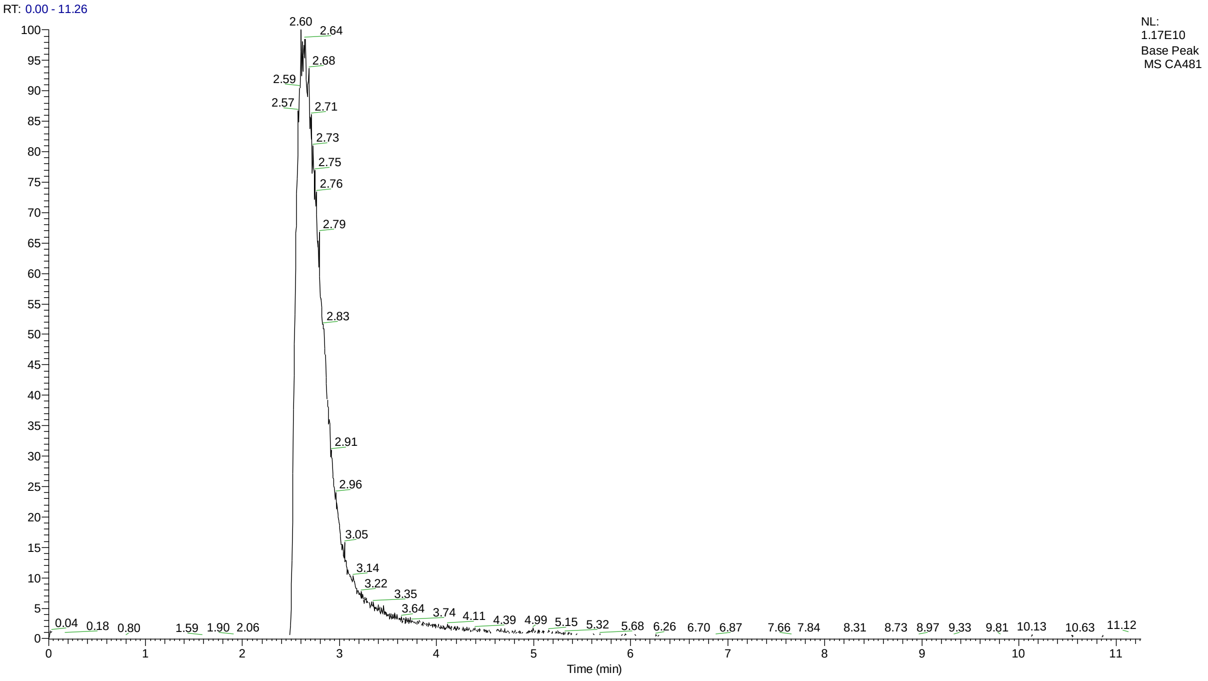

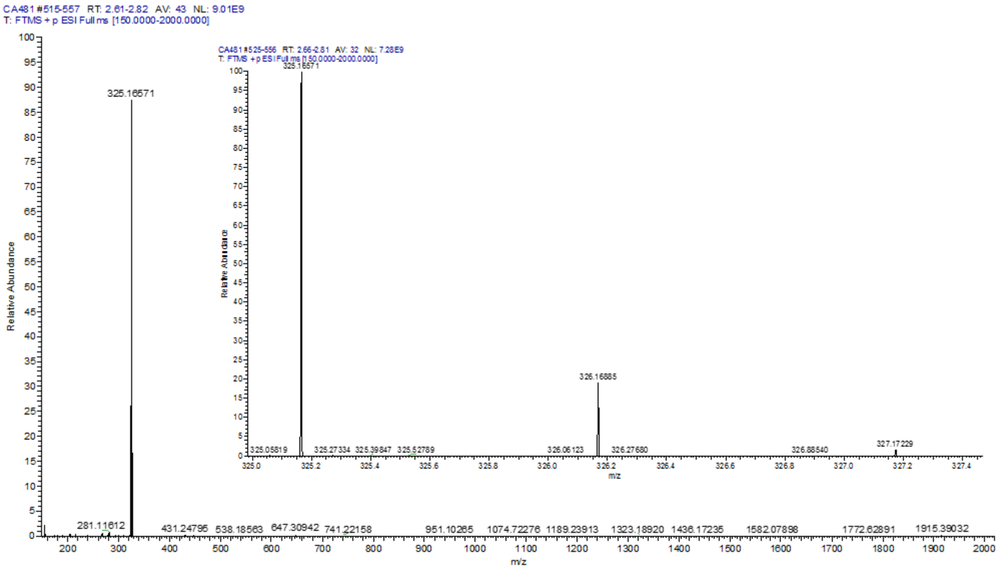

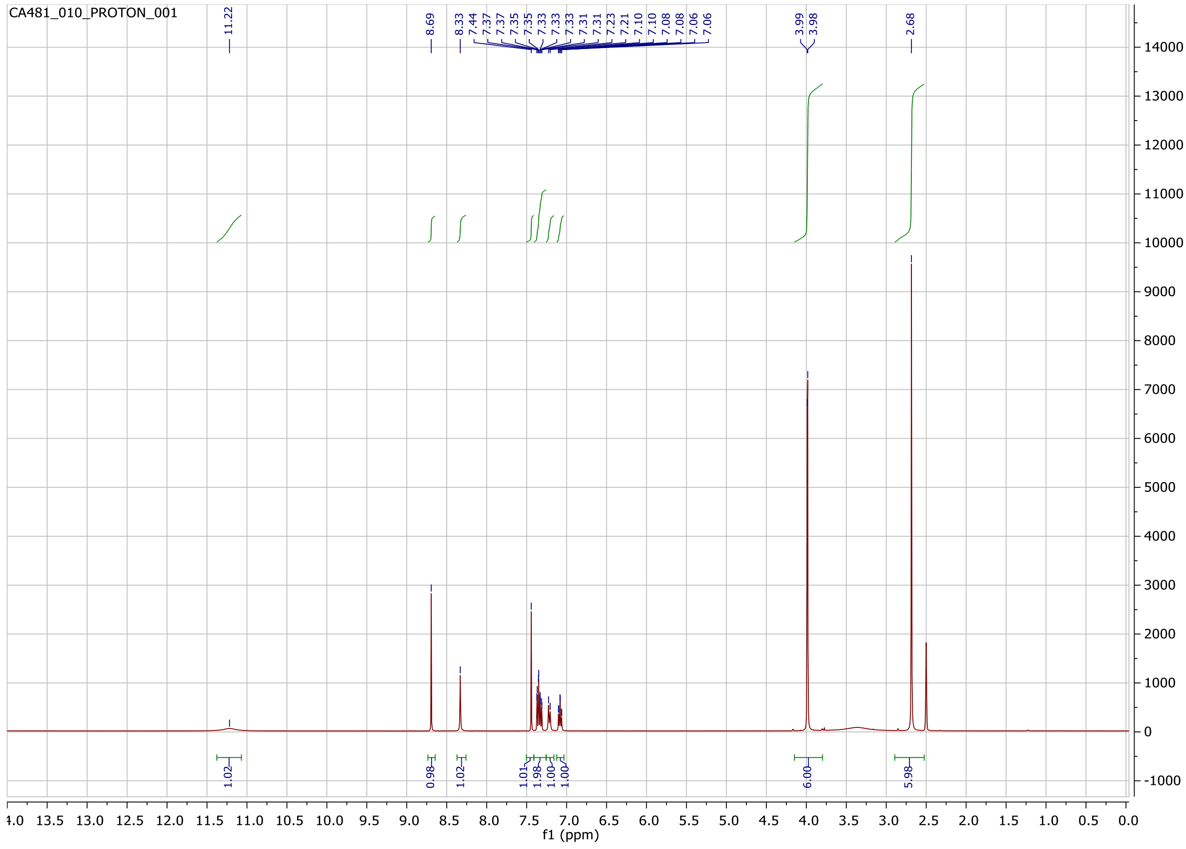
CA481_010_PROTON_001
11.22
8.69
8.33
7.44
7.37
7.37
7.35
7.35
7.33
7.33
7.33
7.31
7.31
7.23
7.21
7.10
7.10
7.08
7.08
7.06
7.06
3.99
3.98
2.68
1.02
0.98
1.02
1.01
1.98
1.00
1.00
6.00
5.98
f1 (ppm)

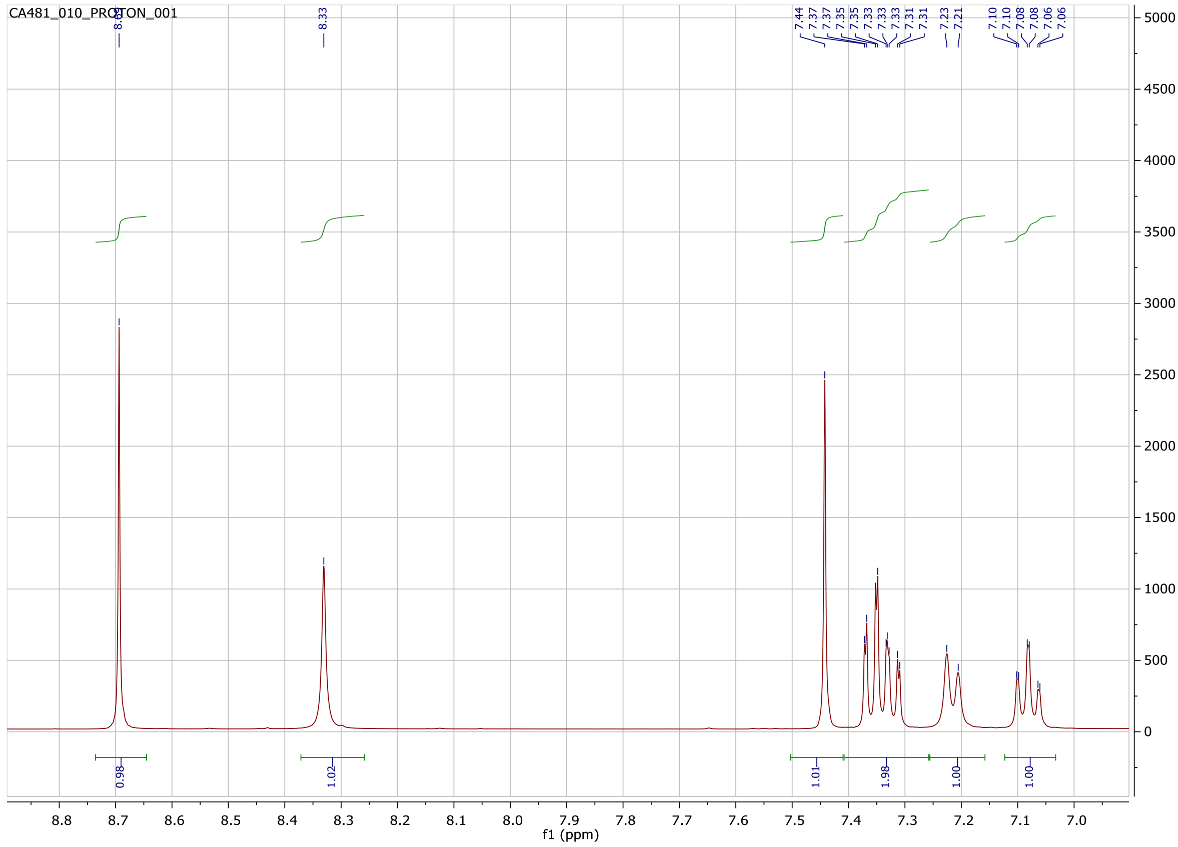
CA481_010_PROTON_001
8.33
7.44
7.37
7.37
7.35
7.35
7.33
7.33
7.33
7.31
7.31
7.23
7.21
7.10
7.10
7.08
7.08
7.06
7.06
0.98
1.02
1.01
1.98
1.00
1.00
f1 (ppm)

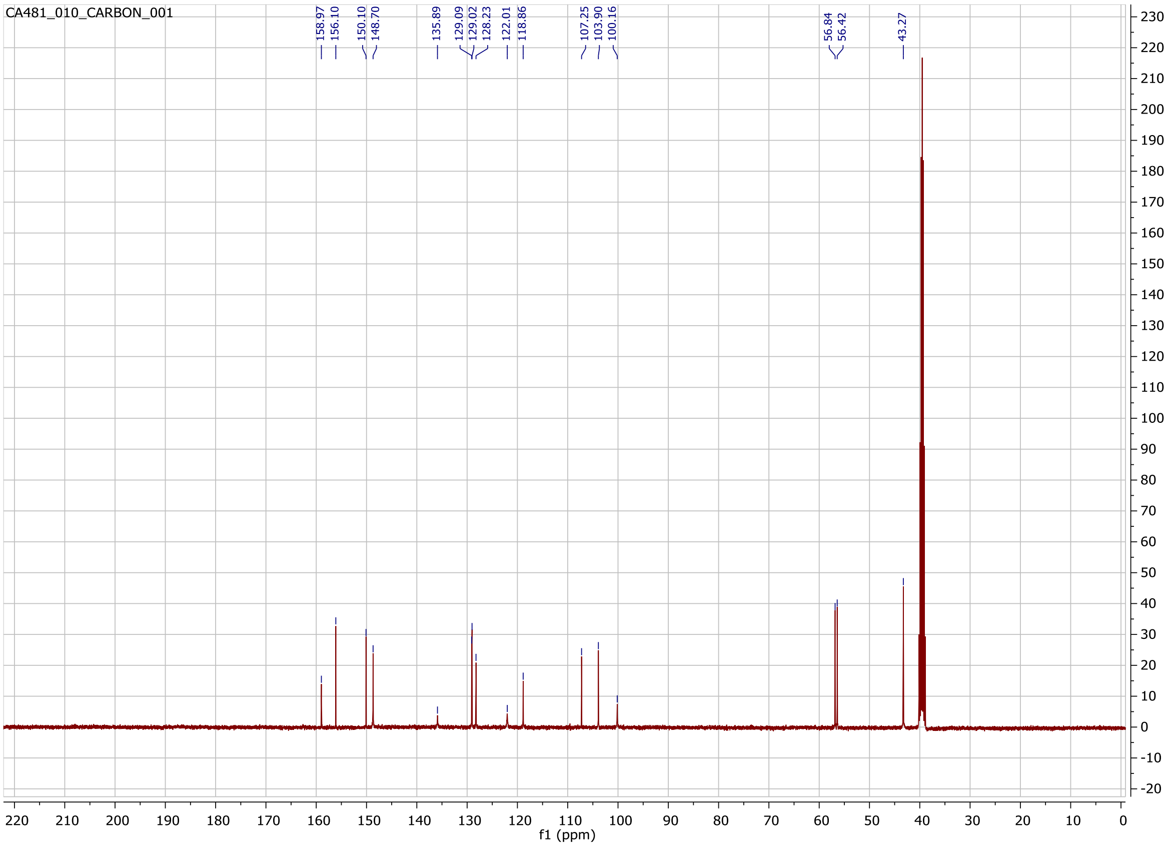

2. LabBook Codes and SMILES for 2, 7, 15-17, 20

Table S1. Lab book numbers and Smiles

Number	Name	Smiles
**2**	CA408	CNS(C1=CC(NC2=C3C(C=C(OC)C(OC)=C3)=NC=N2)=CC=C1)(=O)=O
**7**	CA471	CN(C1=C(NC2=C3C(C=C(C(OC)=C3)OC)=NC=N2)C=C(S(NC)(=O)=O)C=C1)C
**15**	CA472	CN(C1=C(NC2=C3C(C=C(OC)C(OC)=C3)=NC(C)=N2)C=C(S(NC)(=O)=O)C=C1)C
**16**	CA475	CN(C1=C(NC2=C3C(C=CC(OC)=C3)=NC=C2)C=C(S(NC)(=O)=O)C=C1)C
**17**	CA479	CN(C)C(C=CC(S(NC)(=O)=O)=C1)=C1NC2=C3C(C=C(OC)C=C3)=NC=N2
**20**	CA481	CN(C1=C(NC2=C3C(C=C(OC)C(OC)=C3)=NC=N2)C=CC=C1)C

3. KINOMEscan® results of 7

Table S2. KINOMEscan® results of GSK114 (**7**)

DiscoveRx	Entrez	**7** at 1 μM
Gene Symbol		% inhibition
KIT(D816H)	KIT	100
MEK5	MAP2K5	100
PFCDPK1(P.falciparum)	CDPK1	96.3
STK36	STK36	94

ZAK	ZAK	93.1
GAK	GAK	92.1
FLT3(D835V)	FLT3	91.8
KIT(D816V)	KIT	91.7
PDGFRB	PDGFRB	91.4
BRAF	BRAF	84
BRAF(V600E)	BRAF	83
ABL1(Q252H)-phosphorylated	ABL1	79
PIK3C2B	PIK3C2B	77
ABL1(H396P)-nonphosphorylated	ABL1	73
YSK4	MAP3K19	72
PDGFRA	PDGFRA	69
SIK2	SIK2	69
IKK-alpha	CHUK	68
IKK-beta	IKBKB	68
IRAK1	IRAK1	68
GRK2	ADRBK1	65
LCK	LCK	63
TNNI3K	TNNI3K	62
WNK1	WNK1	62
KIT(A829P)	KIT	61
PIK3CD	PIK3CD	61
IRAK4	IRAK4	56
PIK3CA(I800L)	PIK3CA	56
BTK	BTK	54
PAK3	PAK3	54
ZAP70	ZAP70	53
QSK	KIAA0999	52
PIK3CA	PIK3CA	51
TAK1	MAP3K7	51
ABL1(Q252H)-nonphosphorylated	ABL1	50
ADCK3	CABC1	50
KIT(L576P)	KIT	49
RIPK5	DSTYK	49
CAMK1B	PNCK	46
PIK3CG	PIK3CG	45
LOK	STK10	44
LTK	LTK	44
MKK7	MAP2K7	42
DDR2	DDR2	41
MAP4K2	MAP4K2	41
SRMS	SRMS	41
ABL1(F317I)-phosphorylated	ABL1	40
CSF1R-autoinhibited	CSF1R	39
ABL1(Y253F)-phosphorylated	ABL1	38
GRK3	ADRBK2	38

CDK4-cyclinD3	CDK4	37
MEK4	MAP2K4	37
TNK1	TNK1	37
PIK3CA(Q546K)	PIK3CA	36
MEK1	MAP2K1	35
ABL1(E255K)-phosphorylated	ABL1	34
ABL1(H396P)-phosphorylated	ABL1	34
BMPR1B	BMPR1B	34
FLT3(ITD,D835V)	FLT3	34
RSK4(Kin.Dom.1-N-terminal)	RPS6KA6	34
ABL1(T315I)-nonphosphorylated	ABL1	33
MET(Y1235D)	MET	33
PIP5K1C	PIP5K1C	33
RSK2(Kin.Dom.2-C-terminal)	RPS6KA3	33
ERBB3	ERBB3	31
FLT3(R834Q)	FLT3	31
NIM1	MGC42105	31
ASK2	MAP3K6	30
MAP3K3	MAP3K3	30
ABL1(F317L)-nonphosphorylated	ABL1	29
BLK	BLK	29
EGFR(S752-I759del)	EGFR	29
PIK3CA(M1043I)	PIK3CA	29
SBK1	SBK1	29
TAOK3	TAOK3	29
ULK3	ULK3	29
VRK2	VRK2	29
KIT(V559D)	KIT	28
PRKCQ	PRKCQ	28
RIPK2	RIPK2	28
BRSK2	BRSK2	27
MAPKAPK5	MAPKAPK5	27
ABL1-phosphorylated	ABL1	26
TNK2	TNK2	26
VEGFR2	KDR	26
EGFR(E746-A750del)	EGFR	25
EGFR(T790M)	EGFR	25
MEK2	MAP2K2	25
NEK5	NEK5	25
TAOK1	TAOK1	25
TRPM6	TRPM6	25
NLK	NLK	24
PRKCD	PRKCD	24
PRP4	PRPF4B	24
RSK4(Kin.Dom.2-C-terminal)	RPS6KA6	24
GRK7	GRK7	23
LRRK2(G2019S)	LRRK2	23

LZK	MAP3K13	23
PKNB(M.tuberculosis)	pknB	23
RAF1	RAF1	23
S6K1	RPS6KB1	23
CSF1R	CSF1R	22
FLT3(ITD,F691L)	FLT3	22
KIT-autoinhibited	KIT	22
OSR1	OXSR1	22
TRKA	NTRK1	22
ABL1-nonphosphorylated	ABL1	21
DLK	MAP3K12	21
MST1	STK4	21
MST4	MST4	21
CDK4-cyclinD1	CDK4	20
FGFR1	FGFR1	20
FLT3(D835H)	FLT3	20
JAK1(JH2domain-pseudokinase)	JAK1	20
PIK3C2G	PIK3C2G	20
PRKD1	PRKD1	20
TAOK2	TAOK2	20
ANKK1	ANKK1	19
ULK2	ULK2	19
CLK3	CLK3	18
IKK-epsilon	IKBKE	18
MINK	MINK1	18
ULK1	ULK1	18
VPS34	PIK3C3	18
INSR	INSR	17
JAK3(JH1domain-catalytic)	JAK3	17
MELK	MELK	17
PIK3CA(E542K)	PIK3CA	17
PIK3CA(H1047Y)	PIK3CA	17
SIK	SIK1	17
YANK1	STK32A	17
AURKA	AURKA	16
DCAMKL3	DCLK3	15
EPHB6	EPHB6	15
MLK2	MAP3K10	15
SRPK2	SRPK2	15
PIK4CB	PI4KB	14
PRKG2	PRKG2	14
SNRK	SNRK	14
TRKB	NTRK2	14
CLK4	CLK4	13
CSNK2A2	CSNK2A2	13
ERK8	MAPK15	13
FLT3(D835Y)	FLT3	13

HIPK4	HIPK4	13
INSRR	INSRR	13
KIT	KIT	13
MKNK2	MKNK2	13
PCTK1	CDK16	13
PLK2	PLK2	13
PRKCH	PRKCH	13
RSK2(Kin.Dom.1-N-terminal)	RPS6KA3	13
AMPK-alpha2	PRKAA2	12
AXL	AXL	12
DCAMKL1	DCLK1	12
FLT3-autoinhibited	FLT3	12
MAP3K2	MAP3K2	12
NEK4	NEK4	12
CAMK2B	CAMK2B	11
CHEK2	CHEK2	11
CSNK1G3	CSNK1G3	11
HCK	HCK	11
PKN2	PKN2	11
RET	RET	11
ALK	ALK	10
MAST1	MAST1	10
MKNK1	MKNK1	10
MLK1	MAP3K9	10
CAMK2A	CAMK2A	9
CDKL5	CDKL5	9
DAPK3	DAPK3	9
JAK2(JH1domain-catalytic)	JAK2	9
LRRK2	LRRK2	9
MYLK	MYLK	9
NDR2	STK38L	9
RPS6KA4(Kin.Dom.2-C-terminal)	RPS6KA4	9
BUB1	BUB1	8
CLK1	CLK1	8
EGFR(L858R)	EGFR	8
FAK	PTK2	8
JNK1	MAPK8	8
KIT(V559D,T670I)	KIT	8
PRKD2	PRKD2	8
STK39	STK39	8
CLK2	CLK2	7
EGFR(L858R,T790M)	EGFR	7
FLT3	FLT3	7
FLT3(K663Q)	FLT3	7
GCN2(Kin.Dom.2,S808G)	EIF2AK4	7
ICK	ICK	7
MARK1	MARK1	7

PAK6	PAK6	7
PIKFYVE	PIKFYVE	7
PLK4	PLK4	7
PRKD3	PRKD3	7
RPS6KA5(Kin.Dom.1-N-terminal)	RPS6KA5	7
TNIK	TNIK	7
TTK	TTK	7
ABL1(M351T)-phosphorylated	ABL1	6
ACVR2B	ACVR2B	6
CDK7	CDK7	6
ERK5	MAPK7	6
PFPK5(P.falciparum)	MAL13P1.279	6
PKAC-alpha	PRKACA	6
STK33	STK33	6
ALK(L1196M)	ALK	5
CAMKK1	CAMKK1	5
EPHA3	EPHA3	5
HIPK1	HIPK1	5
PIK3CA(C420R)	PIK3CA	5
PIK3CA(E545K)	PIK3CA	5
PRKCI	PRKCI	5
YSK1	STK25	5
MAP3K15	MAP3K15	4
MEK3	MAP2K3	4
RPS6KA4(Kin.Dom.1-N-terminal)	RPS6KA4	4
HASPIN	GSG2	3
MET	MET	3
MST1R	MST1R	3
MARK4	MARK4	2
NEK2	NEK2	2
PIK3CB	PIK3CB	2
RSK1(Kin.Dom.1-N-terminal)	RPS6KA1	2
WNK3	WNK3	2
ABL1(F317I)-nonphosphorylated	ABL1	1
CDK3	CDK3	1
CTK	MATK	1
ROCK1	ROCK1	1
SGK	SGK1	1
SGK3	SGK3	1
AAK1	AAK1	0
ABL1(F317L)-phosphorylated	ABL1	0
ABL1(T315I)-phosphorylated	ABL1	0
ABL2	ABL2	0
ACVR1	ACVR1	0
ACVR1B	ACVR1B	0
ACVR2A	ACVR2A	0
ACVRL1	ACVRL1	0

ADCK4	ADCK4	0
AKT1	AKT1	0
AKT2	AKT2	0
AKT3	AKT3	0
ALK(C1156Y)	ALK	0
AMPK-alpha1	PRKAA1	0
ARK5	NUAK1	0
ASK1	MAP3K5	0
AURKB	AURKB	0
AURKC	AURKC	0
BIKE	BMP2K	0
BMPR1A	BMPR1A	0
BMPR2	BMPR2	0
BMX	BMX	0
BRK	PTK6	0
BRSK1	BRSK1	0
CAMK1	CAMK1	0
CAMK1D	CAMK1D	0
CAMK1G	CAMK1G	0
CAMK2D	CAMK2D	0
CAMK2G	CAMK2G	0
CAMK4	CAMK4	0
CAMKK2	CAMKK2	0
CASK	CASK	0
CDC2L1	CDK11B	0
CDC2L2	CDC2L2	0
CDC2L5	CDK13	0
CDK11	CDK19	0
CDK2	CDK2	0
CDK4	CDK4	0
CDK5	CDK5	0
CDK8	CDK8	0
CDK9	CDK9	0
CDKL1	CDKL1	0
CDKL2	CDKL2	0
CDKL3	CDKL3	0
CHEK1	CHEK1	0
CIT	CIT	0
CSK	CSK	0
CSNK1A1	CSNK1A1	0
CSNK1A1L	CSNK1A1L	0
CSNK1D	CSNK1D	0
CSNK1E	CSNK1E	0
CSNK1G1	CSNK1G1	0
CSNK1G2	CSNK1G2	0
CSNK2A1	CSNK2A1	0
DAPK1	DAPK1	0

DAPK2	DAPK2	0
DCAMKL2	DCLK2	0
DDR1	DDR1	0
DMPK	DMPK	0
DMPK2	CDC42BPG	0
DRAK1	STK17A	0
DRAK2	STK17B	0
DYRK1A	DYRK1A	0
DYRK1B	DYRK1B	0
DYRK2	DYRK2	0
EGFR	EGFR	0
EGFR(G719C)	EGFR	0
EGFR(G719S)	EGFR	0
EGFR(L747-E749del, A750P)	EGFR	0
EGFR(L747-S752del, P753S)	EGFR	0
EGFR(L747-T751del,Sins)	EGFR	0
EGFR(L861Q)	EGFR	0
EIF2AK1	EIF2AK1	0
EPHA1	EPHA1	0
EPHA2	EPHA2	0
EPHA4	EPHA4	0
EPHA5	EPHA5	0
EPHA6	EPHA6	0
EPHA7	EPHA7	0
EPHA8	EPHA8	0
EPHB1	EPHB1	0
EPHB2	EPHB2	0
EPHB3	EPHB3	0
EPHB4	EPHB4	0
ERBB2	ERBB2	0
ERBB4	ERBB4	0
ERK1	MAPK3	0
ERK2	MAPK1	0
ERK3	MAPK6	0
ERK4	MAPK4	0
ERN1	ERN1	0
FER	FER	0
FES	FES	0
FGFR2	FGFR2	0
FGFR3	FGFR3	0
FGFR3(G697C)	FGFR3	0
FGFR4	FGFR4	0
FGR	FGR	0
FLT1	FLT1	0
FLT3(ITD)	FLT3	0
FLT3(N841I)	FLT3	0
FLT4	FLT4	0

FRK	FRK	0
FYN	FYN	0
GRK1	GRK1	0
GRK4	GRK4	0
GSK3A	GSK3A	0
GSK3B	GSK3B	0
HIPK2	HIPK2	0
HIPK3	HIPK3	0
HPK1	MAP4K1	0
HUNK	HUNK	0
IGF1R	IGF1R	0
IRAK3	IRAK3	0
ITK	ITK	0
JAK1(JH1domain-catalytic)	JAK1	0
JNK2	MAPK9	0
JNK3	MAPK10	0
KIT(V559D,V654A)	KIT	0
LATS1	LATS1	0
LATS2	LATS2	0
LIMK1	LIMK1	0
LIMK2	LIMK2	0
LKB1	STK11	0
LYN	LYN	0
MAK	MAK	0
MAP3K1	MAP3K1	0
MAP3K4	MAP3K4	0
MAP4K3	MAP4K3	0
MAP4K4	MAP4K4	0
MAP4K5	MAP4K5	0
MAPKAPK2	MAPKAPK2	0
MARK2	MARK2	0
MARK3	MARK3	0
MEK6	MAP2K6	0
MERTK	MERTK	0
MET(M1250T)	MET	0
MLCK	MYLK3	0
MLK3	MAP3K11	0
MRCKA	CDC42BPA	0
MRCKB	CDC42BPB	0
MST2	STK3	0
MST3	STK24	0
MTOR	MTOR	0
MUSK	MUSK	0
MYLK2	MYLK2	0
MYLK4	MYLK4	0
MYO3A	MYO3A	0
MYO3B	MYO3B	0

NDR1	STK38	0
NEK1	NEK1	0
NEK10	NEK10	0
NEK11	NEK11	0
NEK3	NEK3	0
NEK6	NEK6	0
NEK7	NEK7	0
NEK9	NEK9	0
NIK	MAP3K14	0
p38-alpha	MAPK14	0
p38-beta	MAPK11	0
p38-delta	MAPK13	0
p38-gamma	MAPK12	0
PAK1	PAK1	0
PAK2	PAK2	0
PAK4	PAK4	0
PAK7	PAK7	0
PCTK2	CDK17	0
PCTK3	CDK18	0
PDPK1	PDPK1	0
PFTAIRE2	CDK15	0
PFTK1	CDK14	0
PHKG1	PHKG1	0
PHKG2	PHKG2	0
PIK3CA(E545A)	PIK3CA	0
PIK3CA(H1047L)	PIK3CA	0
PIM1	PIM1	0
PIM2	PIM2	0
PIM3	PIM3	0
PIP5K1A	PIP5K1A	0
PIP5K2B	PIP4K2B	0
PIP5K2C	PIP4K2C	0
PKAC-beta	PRKACB	0
PKMYT1	PKMYT1	0
PKN1	PKN1	0
PLK1	PLK1	0
PLK3	PLK3	0
PRKCE	PRKCE	0
PRKG1	PRKG1	0
PRKR	EIF2AK2	0
PRKX	PRKX	0
PYK2	PTK2B	0
RET(M918T)	RET	0
RET(V804L)	RET	0
RET(V804M)	RET	0
RIOK1	RIOK1	0
RIOK2	RIOK2	0

RIOK3	RIOK3	0
RIPK1	RIPK1	0
RIPK4	RIPK4	0
ROCK2	ROCK2	0
ROS1	ROS1	0
RPS6KA5(Kin.Dom.2-C-terminal)	RPS6KA5	0
RSK1(Kin.Dom.2-C-terminal)	RPS6KA1	0
RSK3(Kin.Dom.1-N-terminal)	RPS6KA2	0
RSK3(Kin.Dom.2-C-terminal)	RPS6KA2	0
SgK110	SgK110	0
SGK2	SGK2	0
SLK	SLK	0
SNARK	NUAK2	0
SRC	SRC	0
SRPK1	SRPK1	0
SRPK3	SRPK3	0
STK16	STK16	0
STK35	STK35	0
SYK	SYK	0
TBK1	TBK1	0
TEC	TEC	0
TESK1	TESK1	0
TGFBR1	TGFBR1	0
TGFBR2	TGFBR2	0
TIE1	TIE1	0
TIE2	TEK	0
TLK1	TLK1	0
TLK2	TLK2	0
TRKC	NTRK3	0
TSSK1B	TSSK1B	0
TSSK3	TSSK3	0
TXK	TXK	0
TYK2(JH1domain-catalytic)	TYK2	0
TYK2(JH2domain-pseudokinase)	TYK2	0
TYRO3	TYRO3	0
WEE1	WEE1	0
WEE2	WEE2	0
WNK2	WNK2	0
WNK4	WNK4	0
YANK2	STK32B	0
YANK3	STK32C	0
YES	YES1	0

4. KINOMEscan® results of 15

Table S3. KINOMEscan® results of **15**

DiscoveRx	Entrez	**15** at 1 µM
Gene Symbol		% inhibition
MEK5	MAP2K5	90.3
GAK	GAK	78
PIK3C2B	PIK3C2B	71
CDK3	CDK3	56
PRP4	PRPF4B	52
MAP3K3	MAP3K3	44
SBK1	SBK1	44
KIT(A829P)	KIT	42
OSR1	OXSR1	42
SGK	SGK1	39
DDR2	DDR2	38
MKNK2	MKNK2	38
PIK3CA(I800L)	PIK3CA	38
FLT3(D835V)	FLT3	37
IKK-beta	IKBKB	37
PIK3CA(Q546K)	PIK3CA	37
PIK3CD	PIK3CD	37
RIPK1	RIPK1	37
TRKA	NTRK1	37
CDK4-cyclinD1	CDK4	36
TNK1	TNK1	35
ABL1(Q252H)-phosphorylated	ABL1	34
ROCK1	ROCK1	34
ABL1(F317L)-nonphosphorylated	ABL1	33
ABL1(Q252H)-nonphosphorylated	ABL1	33
IKK-alpha	CHUK	33
CSF1R-autoinhibited	CSF1R	32
FLT3(R834Q)	FLT3	32
GRK3	ADRBK2	32
ABL1(H396P)-nonphosphorylated	ABL1	31
BMPR1B	BMPR1B	31
EGFR(E746-A750del)	EGFR	31
ULK1	ULK1	31
MKK7	MAP2K7	30
RSK4(Kin.Dom.1-N-terminal)	RPS6KA6	30
CAMK1B	PNCK	28
HIPK3	HIPK3	28
MAP3K15	MAP3K15	28
TSSK3	TSSK3	28
EPHA7	EPHA7	27

MAP3K1	MAP3K1	27
NIM1	MGC42105	27
PFCDPK1(P.falciparum)	CDPK1	27
BTK	BTK	26
CASK	CASK	26
FLT3(ITD,D835V)	FLT3	26
JAK1(JH2domain-pseudokinase)	JAK1	26
EGFR(T790M)	EGFR	25
MEK4	MAP2K4	25
PIK3CB	PIK3CB	25
PKNB(M.tuberculosis)	pknB	25
PRKCI	PRKCI	25
VPS34	PIK3C3	25
ANKK1	ANKK1	24
BRAF(V600E)	BRAF	24
PFPK5(P.falciparum)	MAL13P1.279	24
TAK1	MAP3K7	24
CDC2L5	CDK13	23
CDKL3	CDKL3	23
MAPKAPK5	MAPKAPK5	23
PLK2	PLK2	23
PLK4	PLK4	23
ULK3	ULK3	23
ABL1(F317I)-phosphorylated	ABL1	22
INSRR	INSRR	22
MEK3	MAP2K3	22
MINK	MINK1	22
PCTK1	CDK16	22
PIK3C2G	PIK3C2G	22
PIK3CA(E542K)	PIK3CA	22
PIP5K1A	PIP5K1A	22
QSK	KIAA0999	22
TRKB	NTRK2	22
TRKC	NTRK3	22
WNK1	WNK1	22
BMPR2	BMPR2	21
BRSK2	BRSK2	21
CDK4	CDK4	21
PIK3CA(E545K)	PIK3CA	21
PIK3CA(H1047L)	PIK3CA	21
PIK3CA(H1047Y)	PIK3CA	21
PRKG2	PRKG2	21
BRAF	BRAF	20
GRK1	GRK1	20
S6K1	RPS6KB1	20
CDKL5	CDKL5	19
KIT-autoinhibited	KIT	19

SGK3	SGK3	19
ABL1(Y253F)-phosphorylated	ABL1	18
CDK7	CDK7	18
CSNK2A1	CSNK2A1	18
ERK3	MAPK6	18
MAP3K2	MAP3K2	18
MEK1	MAP2K1	18
PIP5K1C	PIP5K1C	18
PIP5K2C	PIP4K2C	18
SNARK	NUAK2	18
ABL1(F317I)-nonphosphorylated	ABL1	17
DYRK1B	DYRK1B	17
GSK3B	GSK3B	17
p38-delta	MAPK13	17
p38-gamma	MAPK12	17
SRMS	SRMS	17
EGFR(L858R,T790M)	EGFR	16
ERBB3	ERBB3	16
KIT(D816H)	KIT	16
LZK	MAP3K13	16
PRKCQ	PRKCQ	16
RPS6KA4(Kin.Dom.2-C-terminal)	RPS6KA4	16
RSK2(Kin.Dom.2-C-terminal)	RPS6KA3	16
SRPK2	SRPK2	16
TAOK3	TAOK3	16
CDK4-cyclinD3	CDK4	15
DYRK1A	DYRK1A	15
MYO3B	MYO3B	15
PRKD1	PRKD1	15
RSK2(Kin.Dom.1-N-terminal)	RPS6KA3	15
TAOK1	TAOK1	15
TAOK2	TAOK2	15
AURKA	AURKA	14
CSNK2A2	CSNK2A2	14
JNK1	MAPK8	14
LATS1	LATS1	14
LATS2	LATS2	14
MAST1	MAST1	14
PAK2	PAK2	14
WNK2	WNK2	14
ABL1-nonphosphorylated	ABL1	13
CDK11	CDK19	13
CHEK2	CHEK2	13
GRK7	GRK7	13
MST4	MST4	13
NDR1	STK38	13
NEK4	NEK4	13

PIK3CA	PIK3CA	13
PIKFYVE	PIKFYVE	13
PRKD2	PRKD2	13
SIK	SIK1	13
SNRK	SNRK	13
TEC	TEC	13
YSK1	STK25	13
ALK(L1196M)	ALK	12
DLK	MAP3K12	12
ICK	ICK	12
LTK	LTK	12
NEK11	NEK11	12
NIK	MAP3K14	12
PIK4CB	PI4KB	12
RIPK5	DSTYK	12
VEGFR2	KDR	12
VRK2	VRK2	12
EGFR(L747-S752del, P753S)	EGFR	11
INSR	INSR	11
IRAK1	IRAK1	11
PIK3CA(C420R)	PIK3CA	11
PRKCE	PRKCE	11
RSK3(Kin.Dom.1-N-terminal)	RPS6KA2	11
TIE1	TIE1	11
ALK	ALK	10
BMX	BMX	10
FGFR1	FGFR1	10
FLT3	FLT3	10
FLT3(D835H)	FLT3	10
FLT3(K663Q)	FLT3	10
GRK2	ADRBK1	10
JAK3(JH1domain-catalytic)	JAK3	10
KIT	KIT	10
MEK2	MAP2K2	10
PDGFRA	PDGFRA	10
PDGFRB	PDGFRB	10
ABL1(T315I)-nonphosphorylated	ABL1	9
ADCK3	CABC1	9
AXL	AXL	9
DCAMKL1	DCLK1	9
EGFR(S752-I759del)	EGFR	9
HIPK2	HIPK2	9
LIMK2	LIMK2	9
MAP4K4	MAP4K4	9
MST1R	MST1R	9
RIOK2	RIOK2	9
TLK1	TLK1	9

TSSK1B	TSSK1B	9
ULK2	ULK2	9
GRK4	GRK4	8
LRRK2(G2019S)	LRRK2	8
MAK	MAK	8
PLK3	PLK3	8
RIPK4	RIPK4	8
RPS6KA5(Kin.Dom.2-C-terminal)	RPS6KA5	8
TGFBR2	TGFBR2	8
BUB1	BUB1	7
CDC2L1	CDK11B	7
DAPK1	DAPK1	7
HIPK1	HIPK1	7
LOK	STK10	7
PRKR	EIF2AK2	7
TNNI3K	TNNI3K	7
WEE1	WEE1	7
DCAMKL3	DCLK3	6
NDR2	STK38L	6
TIE2	TEK	6
TRPM6	TRPM6	6
CLK4	CLK4	5
DMPK2	CDC42BPG	5
ERK5	MAPK7	5
MST1	STK4	5
PFTAIRE2	CDK15	5
PKN1	PKN1	5
ROCK2	ROCK2	5
RSK1(Kin.Dom.2-C-terminal)	RPS6KA1	5
TXK	TXK	5
AAK1	AAK1	4
ABL1(E255K)-phosphorylated	ABL1	4
AKT3	AKT3	4
ALK(C1156Y)	ALK	4
CAMK2A	CAMK2A	4
CDKL2	CDKL2	4
CIT	CIT	4
EGFR(L858R)	EGFR	4
EIF2AK1	EIF2AK1	4
FGFR4	FGFR4	4
FLT3(D835Y)	FLT3	4
HCK	HCK	4
JNK2	MAPK9	4
KIT(D816V)	KIT	4
MKNK1	MKNK1	4
MLCK	MYLK3	4
MST2	STK3	4

PAK3	PAK3	4
RIPK2	RIPK2	4
SIK2	SIK2	4
AURKB	AURKB	3
CAMKK2	CAMKK2	3
CDK5	CDK5	3
DMPK	DMPK	3
EPHA8	EPHA8	3
ERN1	ERN1	3
IRAK3	IRAK3	3
MAP4K2	MAP4K2	3
MAP4K5	MAP4K5	3
MYLK2	MYLK2	3
PAK6	PAK6	3
PIM3	PIM3	3
RPS6KA4(Kin.Dom.1-N-terminal)	RPS6KA4	3
RSK4(Kin.Dom.2-C-terminal)	RPS6KA6	3
AMPK-alpha2	PRKAA2	2
CAMKK1	CAMKK1	2
CDKL1	CDKL1	2
CSK	CSK	2
EPHA3	EPHA3	2
EPHB2	EPHB2	2
NEK7	NEK7	2
PHKG2	PHKG2	2
PIM1	PIM1	2
STK16	STK16	2
STK39	STK39	2
TTK	TTK	2
AMPK-alpha1	PRKAA1	1
CAMK4	CAMK4	1
CHEK1	CHEK1	1
ERBB2	ERBB2	1
FGFR3	FGFR3	1
MRCKB	CDC42BPB	1
NEK1	NEK1	1
NEK2	NEK2	1
PAK1	PAK1	1
PIK3CA(E545A)	PIK3CA	1
PRKCD	PRKCD	1
SRPK3	SRPK3	1
TESK1	TESK1	1
TNIK	TNIK	1
ZAK	ZAK	1
ABL1(F317L)-phosphorylated	ABL1	0
ABL1(H396P)-phosphorylated	ABL1	0
ABL1(M351T)-phosphorylated	ABL1	0

ABL1(T315I)-phosphorylated	ABL1	0
ABL1-phosphorylated	ABL1	0
ABL2	ABL2	0
ACVR1	ACVR1	0
ACVR1B	ACVR1B	0
ACVR2A	ACVR2A	0
ACVR2B	ACVR2B	0
ACVRL1	ACVRL1	0
ADCK4	ADCK4	0
AKT1	AKT1	0
AKT2	AKT2	0
ARK5	NUAK1	0
ASK1	MAP3K5	0
ASK2	MAP3K6	0
AURKC	AURKC	0
BIKE	BMP2K	0
BLK	BLK	0
BMPR1A	BMPR1A	0
BRK	PTK6	0
BRSK1	BRSK1	0
CAMK1	CAMK1	0
CAMK1D	CAMK1D	0
CAMK1G	CAMK1G	0
CAMK2B	CAMK2B	0
CAMK2D	CAMK2D	0
CAMK2G	CAMK2G	0
CDC2L2	CDC2L2	0
CDK2	CDK2	0
CDK8	CDK8	0
CDK9	CDK9	0
CLK1	CLK1	0
CLK2	CLK2	0
CLK3	CLK3	0
CSF1R	CSF1R	0
CSNK1A1	CSNK1A1	0
CSNK1A1L	CSNK1A1L	0
CSNK1D	CSNK1D	0
CSNK1E	CSNK1E	0
CSNK1G1	CSNK1G1	0
CSNK1G2	CSNK1G2	0
CSNK1G3	CSNK1G3	0
CTK	MATK	0
DAPK2	DAPK2	0
DAPK3	DAPK3	0
DCAMKL2	DCLK2	0
DDR1	DDR1	0
DRAK1	STK17A	0

DRAK2	STK17B	0
DYRK2	DYRK2	0
EGFR	EGFR	0
EGFR(G719C)	EGFR	0
EGFR(G719S)	EGFR	0
EGFR(L747-E749del, A750P)	EGFR	0
EGFR(L747-T751del,Sins)	EGFR	0
EGFR(L861Q)	EGFR	0
EPHA1	EPHA1	0
EPHA2	EPHA2	0
EPHA4	EPHA4	0
EPHA5	EPHA5	0
EPHA6	EPHA6	0
EPHB1	EPHB1	0
EPHB3	EPHB3	0
EPHB4	EPHB4	0
EPHB6	EPHB6	0
ERBB4	ERBB4	0
ERK1	MAPK3	0
ERK2	MAPK1	0
ERK4	MAPK4	0
ERK8	MAPK15	0
FAK	PTK2	0
FER	FER	0
FES	FES	0
FGFR2	FGFR2	0
FGFR3(G697C)	FGFR3	0
FGR	FGR	0
FLT1	FLT1	0
FLT3(ITD)	FLT3	0
FLT3(ITD,F691L)	FLT3	0
FLT3(N841I)	FLT3	0
FLT3-autoinhibited	FLT3	0
FLT4	FLT4	0
FRK	FRK	0
FYN	FYN	0
GCN2(Kin.Dom.2,S808G)	EIF2AK4	0
GSK3A	GSK3A	0
HASPIN	GSG2	0
HIPK4	HIPK4	0
HPK1	MAP4K1	0
HUNK	HUNK	0
IGF1R	IGF1R	0
IKK-epsilon	IKBKE	0
IRAK4	IRAK4	0
ITK	ITK	0
JAK1(JH1domain-catalytic)	JAK1	0

JAK2(JH1domain-catalytic)	JAK2	0
JNK3	MAPK10	0
KIT(L576P)	KIT	0
KIT(V559D)	KIT	0
KIT(V559D,T670I)	KIT	0
KIT(V559D,V654A)	KIT	0
LCK	LCK	0
LIMK1	LIMK1	0
LKB1	STK11	0
LRRK2	LRRK2	0
LYN	LYN	0
MAP3K4	MAP3K4	0
MAP4K3	MAP4K3	0
MAPKAPK2	MAPKAPK2	0
MARK1	MARK1	0
MARK2	MARK2	0
MARK3	MARK3	0
MARK4	MARK4	0
MEK6	MAP2K6	0
MELK	MELK	0
MERTK	MERTK	0
MET	MET	0
MET(M1250T)	MET	0
MET(Y1235D)	MET	0
MLK1	MAP3K9	0
MLK2	MAP3K10	0
MLK3	MAP3K11	0
MRCKA	CDC42BPA	0
MST3	STK24	0
MTOR	MTOR	0
MUSK	MUSK	0
MYLK	MYLK	0
MYLK4	MYLK4	0
MYO3A	MYO3A	0
NEK10	NEK10	0
NEK3	NEK3	0
NEK5	NEK5	0
NEK6	NEK6	0
NEK9	NEK9	0
NLK	NLK	0
p38-alpha	MAPK14	0
p38-beta	MAPK11	0
PAK4	PAK4	0
PAK7	PAK7	0
PCTK2	CDK17	0
PCTK3	CDK18	0
PDPK1	PDPK1	0

PFTK1	CDK14	0
PHKG1	PHKG1	0
PIK3CA(M1043I)	PIK3CA	0
PIK3CG	PIK3CG	0
PIM2	PIM2	0
PIP5K2B	PIP4K2B	0
PKAC-alpha	PRKACA	0
PKAC-beta	PRKACB	0
PKMYT1	PKMYT1	0
PKN2	PKN2	0
PLK1	PLK1	0
PRKCH	PRKCH	0
PRKD3	PRKD3	0
PRKG1	PRKG1	0
PRKX	PRKX	0
PYK2	PTK2B	0
RAF1	RAF1	0
RET	RET	0
RET(M918T)	RET	0
RET(V804L)	RET	0
RET(V804M)	RET	0
RIOK1	RIOK1	0
RIOK3	RIOK3	0
ROS1	ROS1	0
RPS6KA5(Kin.Dom.1-N-terminal)	RPS6KA5	0
RSK1(Kin.Dom.1-N-terminal)	RPS6KA1	0
RSK3(Kin.Dom.2-C-terminal)	RPS6KA2	0
SgK110	SgK110	0
SGK2	SGK2	0
SLK	SLK	0
SRC	SRC	0
SRPK1	SRPK1	0
STK33	STK33	0
STK35	STK35	0
STK36	STK36	0
SYK	SYK	0
TBK1	TBK1	0
TGFBR1	TGFBR1	0
TLK2	TLK2	0
TNK2	TNK2	0
TYK2(JH1domain-catalytic)	TYK2	0
TYK2(JH2domain-pseudokinase)	TYK2	0
TYRO3	TYRO3	0
WEE2	WEE2	0
WNK3	WNK3	0
WNK4	WNK4	0
YANK1	STK32A	0

YANK2	STK32B	0
YANK3	STK32C	0
YES	YES1	0
YSK4	MAP3K19	0
ZAP70	ZAP70	0

5. Mass Spectrometry Method

Samples were analyzed with a ThermoFisher Q Exactive HF-X (ThermoFisher, Bremen, Germany) mass spectrometer coupled with a Waters Acquity H-class liquid chromatograph system. Samples were introduced via a heated electrospray source (HESI) at a flow rate of 0.6 mL/min. Electrospray source conditions were set as: spray voltage 3.0 kV, sheath gas (nitrogen) 60 arb, auxiliary gas (nitrogen) 20 arb, sweep gas (nitrogen) 0 arb, nebulizer temperature 375 degrees C, capillary temperature 380 degrees C, RF funnel 45 V. The mass range was set to 150-2000 m/z. All measurements were recorded at a resolution setting of 120,000.

Separations were conducted on a Waters Acquity UPLC BEH C18 column (2.1 x 50 mM, 1.7 μM particle size). LC conditions were set at 100 % water with 0.1 % formic acid (A) ramped linearly over 9.8 mins to 95 % acetonitrile with 0.1 % formic acid (B) and held until 10.2 mins. At 10.21 mins the gradient was switched back to 100% A and allowed to re-equilibrate until 11.25 mins. Injection volume for all samples was 3 μL.

Xcalibur (ThermoFisher, Breman, Germany) was used to analyze the data. Solutions were analyzed at 0.1 mg/mL or less based on responsiveness to the ESI mechanism. Molecular formula assignments were determined with Molecular Formula Calculator (v 1.2.3). All observed species were singly charged, as verified by unit m/z separation between mass spectral peaks corresponding to the ^{12}C and $^{13}C^{12}C_{c-1}$ isotope for each elemental composition.
